# Sustainability management of short-lived freshwater fish in human-altered ecosystems should focus on adult survival

**DOI:** 10.1371/journal.pone.0232872

**Published:** 2020-05-12

**Authors:** Michael D. Hatch, Fitsum Abadi, Wiebke J. Boeing, Sabela Lois, Michael D. Porter, David E. Cowley

**Affiliations:** 1 Department of Fish, Wildlife & Conservation Ecology, New Mexico State University, Las Cruces, New Mexico, United States of America; 2 Water Science & Management Program, New Mexico State University, Las Cruces, New Mexico, United States of America; 3 U. S. Army Corps of Engineers, Albuquerque, New Mexico, United States of America; Universidade Federal de Mato Grosso do Sul, BRAZIL

## Abstract

Fish populations globally are susceptible to endangerment through exploitation and habitat loss. We present theoretical simulations to explore how reduced adult survival (age truncation) might affect short-lived freshwater fish species in human-altered contemporary environments. Our simulations evaluate two hypothetical "average fish" and five example fish species of age 1 or age 2 maturity. From a population equilibrium baseline representing a natural, unaltered environment we impose systematic reductions in adult survival and quantify how age truncation affects the causes of variation in population growth rate. We estimate the relative contributions to population growth rate arising from simulated temporal variation in age-specific vital rates and population structure. At equilibrium and irrespective of example species, population structure (first adult age class) and survival probability of the first two adult age classes are the most important determinants of population growth. As adult survival decreases, the first reproductive age class becomes increasingly important to variation in population growth. All simulated examples show the same general pattern of change with age truncation as known for exploited, longer-lived fish species in marine and freshwater environments. This implies age truncation is a general potential concern for fish biodiversity across life history strategies and ecosystems. Managers of short-lived, freshwater fishes in contemporary environments often focus on supporting reproduction to ensure population persistence. However, a strong focus on water management to support reproduction may reduce adult survival. Sustainability management needs a focus on mitigating adult mortality in human-altered ecosystems. A watershed spatial extent embracing land and water uses may be necessary to identify and mitigate causes of age truncation in freshwater species. Achieving higher adult survival will require paradigm transformations in society and government about water management priorities.

## Introduction

Despite a 450 million-year evolutionary history punctuated with global climate fluctuations and mass extinctions, contemporary biodiversity exceeds 35000 species of fish [1], more than one-half of all vertebrates. However, in the Anthropocene (http://quaternary.stratigraphy.org/working-groups/anthropocene/, accessed 11 February 2020) numerous fish species globally have declined severely from over-exploitation [2] and habitat modification by humans [3]. Continued human population growth should be expected to increase direct impacts on harvested fish stocks and indirect impacts on all fishes with modifications to the waters they occupy. A transition in policy [4] is urgently needed to confront growing human needs for freshwaters while also recognising and mitigating indirect ecological effects on freshwater biota.

Managers of fish species for recreational, subsistence or commercial pursuits commonly regulate harvest aiming to reduce adult mortality, a general affirmation that adult survival is important for sustainable use [5]. In contrast, with nongame fish species the management focus is often on supporting successful reproduction in hopes of facilitating species’ survival [6,7]. A focus on reproduction implies that adult survival may be less important for conservation of endangered freshwater fish species, or that improving reproduction will offset the adult mortality the species experiences in its contemporary environment. Prior work on the relative importance of adult survival for freshwater fish populations is contradictory. Velez-Espino et al. [8] suggested that juvenile survival and fecundity were more important than adult survival for short-lived freshwater fishes. In contrast, Wang et al. [9] reported that population growth was generally more dependent on juvenile and adult survival and reproductive output was of minor importance. Is adult survival important for conservation of short-lived freshwater fishes? We use a matrix population model to explore what happens to a population when adult survival declines.

Reconciling the shortcomings of asymptotic theory of population ecology with the reality of unstable, altered environments occupied by endangered species is a contemporary problem in conservation. The inadequacy of asymptotic theory for endangered species can include probable lack of a stable population structure, survival probabilities that vary across years and erratic habitat conditions that contribute additional mortality in some years [10,11]. Although it relies on a single life history of vital rates, an asymptotic perspective has been commonly applied to conservation including numerous applications to fish conservation [9,12–14]. The asymptotic approach assumes equilibrium conditions with a stable population structure in a stationary environment. Stochastic variation in vital rates driven by fluctuations in the environment is ignored in the asymptotic approach and as a result, an asymptotic approach can fail to identify key factors contributing to population growth rate and can promote ineffective conservation when environmental conditions are variable in time.

It would be naïve to suggest there is a single asymptotic set of vital rates that are valid over all habitat conditions [15] because environmental stochasticity can affect age-specific survival and reproductive potential. Temporal variation in vital rates introduces variation in population structure that can have a large effect on population growth, especially for life histories with low juvenile survival probability [16]. Generally in fishes, juvenile survival rates are low and they vary inversely with fecundity [17]. Although an investigator can obtain sample estimates of survival probabilities for an endangered species, samples in different years or locations are likely to yield different estimates. It can be difficult to know exactly which set of estimates is appropriate for use in an asymptotic analysis of matrix population models. As a consequence, a transient approach may be superior to an asymptotic perspective in addressing the question of the relative importance of adult survival for short-lived fish species. Life table response experiments (LTREs) using controlled perturbations [18] are not likely to be permitted or practical for an endangered species. The inevitable uncertainty in estimates of vital rates for an endangered species led us to use simulated transient LTREs [16] to evaluate the effects of perturbations to vital rates on population growth rate.

Here we use simulations of temporal variation in vital rates and retrospective transient LTREs to explore how fish populations might respond to environmental variation that reduces adult survival. Reduced adult survival can drive rapid change in fish populations with high natural predation [19] or strong exploitation [20]. The manifestation of reduced adult survival in fish populations has been variously called age truncation [21], juvenescence [22], longevity overfishing [23], recruitment overfishing [24,25] and growth overfishing [26]. Irrespective of its cause, age truncation occurs when reduced adult survival probability causes a decline in the mean age of adults and decreases the population’s future reproductive potential [27], while also shortening a species’ effective life span as older adults become rarer.

**Fig 1:**
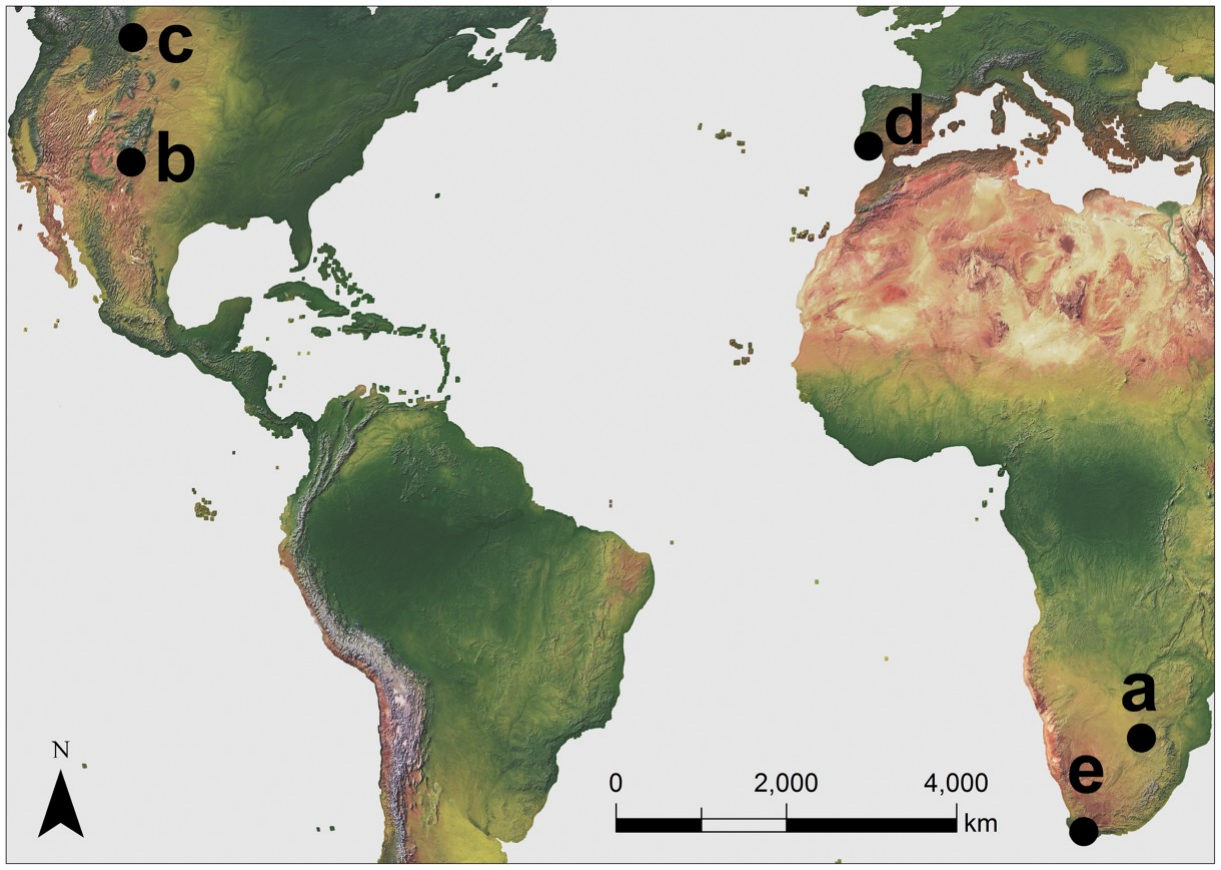
Locations of example species used in simulations of age truncation. (a) *Enteromius motebensis*, Marico River, South Africa; (b) *Hybognathus amarus*, Rio Grande, New Mexico, USA; (c) *Hybognathus argyritis*, Milk River, Alberta, Canada; (d) *Iberochondrostoma lusitanicum*, Torgal Rivulet, Portugal; (e) *Pseudobarbus burchelli*, Breede River, South Africa; Made with Natural Earth. Free vector and raster map data @ naturalearthdata.com.

In this study, we develop a fecundity equation for an iteroparous "average fish" and a hypothetical equilibrium baseline from which to simulate age truncation. For comparative purposes, we further mimic the life history of five freshwater fish species (Order Cypriniformes) [28] from three continents that differ variously in life span, growth rate, maximum size, age at maturity and age-specific fecundity (Fig 1, Table 1): Marico barb (*Enteromius motebensis*; Family Cyprinidae: Smiliogastrinae), Rio Grande silvery minnow (*Hybognathus amarus*; Leuciscidae: Pogonichthyinae), western silvery minnow (*Hybognathus argyritis*; Leuciscidae: Pogonichthyinae), boga portuguesa (*Iberochondrostoma lusitanicum*; Leuciscidae: Leuciscinae) and Burchell’s redfin (*Pseudobarbus burchelli*; Cyprinidae: Smiliogastrinae). These example species are broadly representative of iteroparity, the most common reproductive strategy among ray-finned fishes (Actinopteryi), which comprise about two-thirds of all fish species [1]. We had access to more information for *H*. *amarus*, a local species in our region. The additional example species had sufficient published information to approximate their life history. We chose these species and the comparable-sized hypothetical "average fish" to address the question: "Is there similarity or dissimilarity across different life histories in how the drivers of variation in population growth respond to age truncation in short-lived fish species?"

**Table 1:** A synopsis of study species and data sources.

Species^a^	Loc^b^	Mat^c^	Age^d^	Threats	Status^e^	Refs
*Enteromius motebensis*	SA	1	4+	Pollution, water extractions, exotic predatory fishes	NT	[53,64]
*Hybognathus amarus*	USA	1	4+	Dams, water extractions, exotic predatory fishes, intermittency	E	[52,65–66]
*Hybognathus argyritis*	CA	2	5+	Water extractions, climate change	NT	[67–68]
*Iberochondrostoma lusitanicum*	PT	2	5+	Exotic predatory fishes, intermittency	CE	[54]
*Pseudobarbus burchelli*	SA	2	5+	Exotic predatory fishes	CE	[51]

^a^Species common names given in text

^b^Country locations (Loc) are shown in Fig 1: South Africa (SA), United States of America (USA), Canada (CA), Portugal (PT)

^c^Age (yr) at sexual maturity (Mat)

^d^Approximate life span (yr) assumed in simulations

^e^IUCN categories: near threatened (NT), endangered (E), critically endangered (CE)

## Methods

Terms and symbols used in this paper are defined in (S1 Table). Our approach to study age truncation in short-lived freshwater fishes involved these aspects: (1) development of a theoretical equilibrium population baseline, (2) compilation of published fecundity and body length data for 33 freshwater cypriniform species, and Bayesian meta-analysis to enable prediction of fecundity at-age, (3) simulation of temporal variation in fecundity and survival at four levels of adult survival, (4) retrospective analysis of simulations to infer relative contributions of each vital rate and component of population structure to population growth rate and (5) comparison of simulation results across example species.

### Ethics statement

Laboratory experiments on post-hatch *H*. *amarus* were conducted under permit TE065394-2 (U.S. Fish and Wildlife Service) and with authorization of the New Mexico State University Institutional Animal Care and Use Committee (IACUC, # 2005-006boeing). Field collections of *H*. *amarus* were conducted under permit TE045-235-3 (U.S. Fish and Wildlife Service) and permit 3143 (New Mexico Department of Game and Fish) by the senior author in association with SWCA Environmental Consultants, Albuquerque, New Mexico.

### Implementation of a matrix population model

We use a single matrix population model that accommodates life spans of age 4+ and age 5+. We assume the last age class is composed of age 5 and older individuals, of which there is a non-zero probability of occurrence even with adult survival for an age 2 longevity. An age class model is preferred to a juvenile-adult stage-based model because many fish species have a strongly allometric increase in fecundity with body length (and age) that exceeds the slope of the relationship between fecundity and body mass [29,30]; a stage-based model may not accurately represent the contribution of fecundity from older females with disproportionately higher fecundity.

The vital rate parameters (Eq 1) in the simulated post-breeding birth-pulse female transition matrix include age-specific survival probability (*S*_*i*_) and fecundity (number of eggs) (*F*_*i*_). We assumed density independence because other species with similar life histories have conformed poorly to models of density dependence [31]. Note that the age-specific number of individuals (*n*_*i*_) in the population vector are additional model parameters in transient LTREs [16]. We assume that *S*_1_ = … = *S*_5_ in the starting transition matrix for each simulation scenario; for simulations of delayed maturity *F*_1_ = 0.

$${\left[\begin{array}{r} n_{0}\\ n_{1}\\ n_{2}\\ n_{3}\\ n_{4}\\ n_{5} \end{array}\right]}_{t}={\left[\begin{array}{rrrrrr} 0 & S_{1}F_{1} & S_{2}F_{2} & S_{3}F_{3} & S_{4}F_{4} & S_{5}F_{5}\\ S_{0} & 0 & 0 & 0 & 0 & 0\\ 0 & S_{1} & 0 & 0 & 0 & 0\\ 0 & 0 & S_{2} & 0 & 0 & 0\\ 0 & 0 & 0 & S_{3} & 0 & 0\\ 0 & 0 & 0 & 0 & S_{4} & S_{5} \end{array}\right]}_{t}{\left[\begin{array}{r} n_{0}\\ n_{1}\\ n_{2}\\ n_{3}\\ n_{4}\\ n_{5} \end{array}\right]}_{t-1}$$ (1)

To facilitate simulating age truncation, we develop a hypothetical "natural equilibrium" view of a species in its natural environment wherein adult survival probability determines approximate natural lifespan. We assume for a natural equilibrium that a species’ fecundity at size and age is a result of selection in the natural environment. If juvenile mortality is a stochastic determination of the environment, then a species that persists in the natural environment must have fecundity at-age high enough to compensate for juvenile mortality.

In this hypothetical context, a species would have population growth rate close to equilibrium (λ ≌ 1) and the population would have an equilibrium age-class structure after some generations in the natural environment. A natural equilibrium perspective is helpful for simulations because there is inevitable uncertainty about true values of vital rates in contemporary, perturbed environments, and secondly, it provides a convenient frame of reference that can be developed for any species. With limited data it is possible to approximate aspects of life history sufficiently to define a natural equilibrium baseline for each example species. We simulate age truncation for age 1 and age 2 maturities because some fish species delay reproduction and attain larger body size and reproductive potential. We next describe how we derived values for survival probabilities and fecundity of our study species.

Adult survival. We used a theoretical context to derive the adult survival probability. Prior meta-analysis [32] and application [33] establish that natural mortality (*M*) in fishes is best approximated as *M* = 4.3/*T*_*max*_, where *T*_*max*_ is the species’ life span (years) in an unperturbed natural environment (S2 Fig). The adult survival probability necessary for this longevity is *S*_*1*_ = … = *S*_*5*_ = *e*^*-M*^. For exploited fish populations adult survival probability is commonly estimated as *S*_*1*_ = … = *S*_*5*_ = *e*^*-(E+M)*^, where natural mortality (*M*) is augmented by *E*, which represents mortality from environmental sources.

We begin our simulations at an adult survival necessary for the assumed life span for each species. The estimated values of adult survival using the equation given by [32] for natural mortality are 0.34 and 0.42 for life spans of four and five years, respectively. In our simulations we use adult survival values of 0.35 and 0.45 to represent life spans of ages 4+ and 5+, respectively. From this hypothetical baseline, we simulated systematically at values of adult survival of 0.15, 0.25, 0.35 and 0.45, while holding *S*_*0*_ at its equilibrium value. Note that adult survival values of 0.25 and 0.15 in our simulations are equivalent to age-truncated life histories of age 3+ and 2+, respectively. We calculate a value of *E*, environmental mortality, at each reduced level of adult survival. Notice that *e*^*-(E+M)*^ = *e*^*-E*^ x *e*^*-M*^ where *e*^*-E*^ represents survivorship to environmental mortality and *e*^*-M*^ is survivorship to natural mortality.

#### Age 0 survival (S_0_)

We obtain an equilibrium estimate [34] of the survival probability of age 0 using species-specific values of fecundity at-age and the adult survival necessary for its approximate life span in an unperturbed natural environment. This equilibrium estimate of *S*_*0*_ (S2 Table), given a species’ fecundity at-age and natural life span, yields a stable population at equilibrium with its environment (λ_1_ = 1) as the frame of reference in simulations for each species.

For a comparison with equilibrium estimates of *S*_*0*_, we used previously unpublished data for *H*. *amarus* from a laboratory experiment on larval survival using four replicate microcosms. Additional details are given in (S3 Table) and data are provided in S1 Data. The geometric mean daily survival probability was calculated for each microcosm and an annual estimate of age 0 survival probability was obtained using the geometric mean daily survival probability across replicates.

#### Age-specific fecundity

We used Bayesian meta-analysis of 119 marine and freshwater fish species to estimate fecundity at-age for each species in our simulations. There is compelling evidence for a hyperallometric relationship between fecundity and body size in almost all fish species [30]. We downloaded supporting data [30] for 342 marine species in 15 orders and to this dataset we added fecundity-body length data extracted from published literature for 33 species of freshwater fish (Order Cypriniformes, n = 1359), thus adding a 16th order to the dataset. A list of the cypriniform species is given in (S4 Table) and data are given in S1 Data. Fecundity and length data were extracted from tables or digitized from published figures; thus, data from figures are an approximation of the original values. On a logarithmic scale we assume approximation errors are independent and small.

We inspected a scatterplot of the data (S2 Fig) and elected to use a hierarchical modeling framework to assess the relationship between fecundity (Y) and length (X). Both variables were transformed using a logarithmic scale. In the analysis we excluded species with less than 10 observations, yielding data for 119 species (33 freshwater and 86 marine species) and a total of 7721 observations. We fitted a model with species-specific intercept and a common slope for all species (eq. 2) to predict fecundity:

$$\text{log}\left(Y_{ij}\right)=\beta_{0\left(j\right)}+\beta_{1}\text{log}\left(X_{ij}\right)+\varepsilon_{ij},i=1,2,\ldots ,7721;j=1,2,\ldots ,119$$ (2)

$$\beta_{0(j)}\sim N(\beta_{0},\sigma_{b}^{2})$$

$$\varepsilon_{ij}\sim N(0,\sigma_{\varepsilon }^{2})$$

where *Y*_*ij*_ and *X*_*ij*_ are the fecundity and length of the *i*^*th*^ individual of species *j*, respectively, *β*_0(*j*)_ is the intercept for species *j* (i.e. species-specific intercept), *β*_0_ and *β*_1_ are the mean intercept and common slope, respectively, *ε*_*ij*_ is the residual, and $\sigma_{b}^{2}$ and $\sigma_{\varepsilon }^{2}$ are the variances for the random intercept and residuals, respectively.

We specified non-informative priors for all model parameters (*β*_0_ ~ *Normal*(0, 0.001), *β*_1_ ~ *Normal*(0, 0.001), *σ*_*b*_ ~ *Uniform*(0, 100), *σ*_*ε*_ ~ *Uniform*(0, 100)) and ran three independent MCMC chains of 5000 iterations with a burn-in of 2000 iterations to obtain posterior estimates of model parameters. We assessed convergence using the Brooks-Gelman-Rubin diagnostic statistic ($\hat{R}$) [35] and by visually inspecting the trace plots. The trace plots for all parameters showed a good mixing and the $\hat{R}$ values for all parameters were below 1.1, indicating there was no lack of convergence. We implemented the Bayesian meta-analysis in JAGS [36] using the ’jagsUI' [37] package in R [38].

Results from the Bayesian meta-analysis were used to predict fecundity at mean length at-age for an "average fish" of age 1 or age 2 maturity using the mean intercept and common slope from Bayesian meta-analysis. For each example species we estimated fecundity using the species-specific intercept and common slope. For *E*. *motebensis*, *H*. *argyritis*, *I*. *lusitanisum* and *P*. *burchelli*, we obtained or inferred mean size at-age from published literature. For *H*. *amarus*, mean size at age was estimated from a large sample of *H*. *amarus* (N = 2423) collected in May 2009 [39]; data are provided in S1 Data. Individual age was estimated using a modelled age-length key [40]; additional details are given in (S5 Table).

### Simulation details

Our approach to study age-truncation involved retrospective analysis of simulated transient variation in vital rates and population structure using transient LTREs [16]. For each simulation we generated random values of vital rates for 25 time steps [16]. At each time step, survival probabilities were drawn from a beta distribution with expected value equal to the starting value in the transition matrix and shape parameters *a* and *b* chosen for a coefficient of variation (CV) of 0.2. The effect of different levels of CV were evaluated at CV = 0.05, 0.1, 0.2 and 0.3. Random deviates for fecundity were drawn from a lognormal distribution with expected value equal to the logarithm of predicted fecundity and standard deviation (estimated from Bayesian analysis) equal to the standard deviation of the species-specific intercept divided by the square root of the sample size, an approximate standard error for mean fecundity at-age. Notice that a random transition matrix is generated each simulation time step using the starting transition matrix as the expected values for vital rates.

The stochastic realisation of the transition matrix at each time step (*t*) was used to calculate the realised population growth rate (λ_t_ = *N*_*t*_/*N*_*t-1*_), where population structure (*n*_*i*_) is normalized at time *t-1* [16]. The variance of realised population growth rate ($\sigma_{\lambda t}^{2}$) was decomposed into a proportional contribution for each parameter in the matrix population model using the example of Koons et al. [16]. The estimation of parameter contributions involved calculation of sensitivities to changes in vital rates and population structure, and temporal covariances among these parameters [16]. Each simulation scenario was replicated 100 times and mean contributions were obtained for each vital rate and component of population structure. Transient LTRE results were summarized for each species by plotting the mean proportional contribution for each vital rate contributing at least 10% of $\sigma_{\lambda t}^{2}$ at each level of adult survival. All simulations were conducted in R [38] following the example of Koons et al. [16].

## Results

Values used in simulations for age at maturity and age-specific length (L_i_) and fecundity (*F*_*i*_) are shown in Table 2. Species simulated with age 1 maturity (age 4+ life span) had smaller adult body size and lower fecundity than did the species in simulations of age 2 maturity (age 5+). The *F*_*i*_ shown in Table 2 were calculated using the results from Bayesian meta-analysis of fecundity-body length (S6 Table). The slope (3.447) and mean intercept over all species (-8.101) were used to compute fecundity at-age for a hypothetical "average fish" of age 1 or age 2 maturity, given an approximately scaled length at-age (Table 2). The Bayesian estimates of species-specific intercept (S6 Table) were used to calculate fecundity for each example species (Table 2).

**Table 2:** Age of maturity and values of length and fecundity used in simulations of age truncation.

		Length (mm) at Age					Fecundity (eggs) at Age^a^				
Species	M^b^	L_1_	L_2_	L_3_	L_4_	L_5_	*F*_1_	*F*_2_	*F*_3_	*F*_4_	*F*_5_
"ave. fish"^c^	1	50	65	75	80	85	218	538	881	1101	1357
"ave. fish"^c^	2	45	75	90	108	120	0	881	1652	3097	4454
*E*. *motebensis*	1	50	65	75	80	85	683	1688	2764	3451	4255
*H*. *amarus*^d^	1	51	61	69	74	82	1830	3392	5187	6602	9405
*H*. *argyritis*	2	50	81	105	118	130	0	3273	8008	11975	16721
*I*. *lusitanicum*	2	45	75	95	115	125	0	1505	3399	6567	8753
*P*. *burchelli*	2	45	75	85	100	115	0	1125	1732	3032	4909

^a^Age-specific fecundity was calculated using the overall slope (3.447) and species-specific intercept (S6 Table) from Bayesian meta-analysis.

^b^M denotes age of maturity used in simulation.

^c^Fecundity for "average fish" was calculated using the overall slope (3.447) and mean intercept (-8.101).

^d^Fecundity data were from captive fish, which may or may not be accurate for wild fish.

Equilibrium estimates of *S*_*0*_ (S2 Table) ranged from 4.562 x 10^−4^ to 4.911 x 10^−3^. The equilibrium value of *S*_*0*_ = 7.008 x 10^−4^ for *H*. *amarus* was two orders of magnitude smaller than survival estimated from the laboratory experiment where *S*_*0*_ = 3.046 x 10^−2^ (S3 Table).

In simulations varying the CV for survival, values of CV < 0.2 resulted in slightly greater importance of fecundity (S3 Fig) to variation in population growth rate. However, in all transient LTREs for example species (S4–S10 Figs) fecundity was consistently a minor contributor to population growth. An archive of all simulation scripts and results is available in Dryad (https://doi.org/10.5061/dryad.69p8cz8z7).

**Fig 2:**
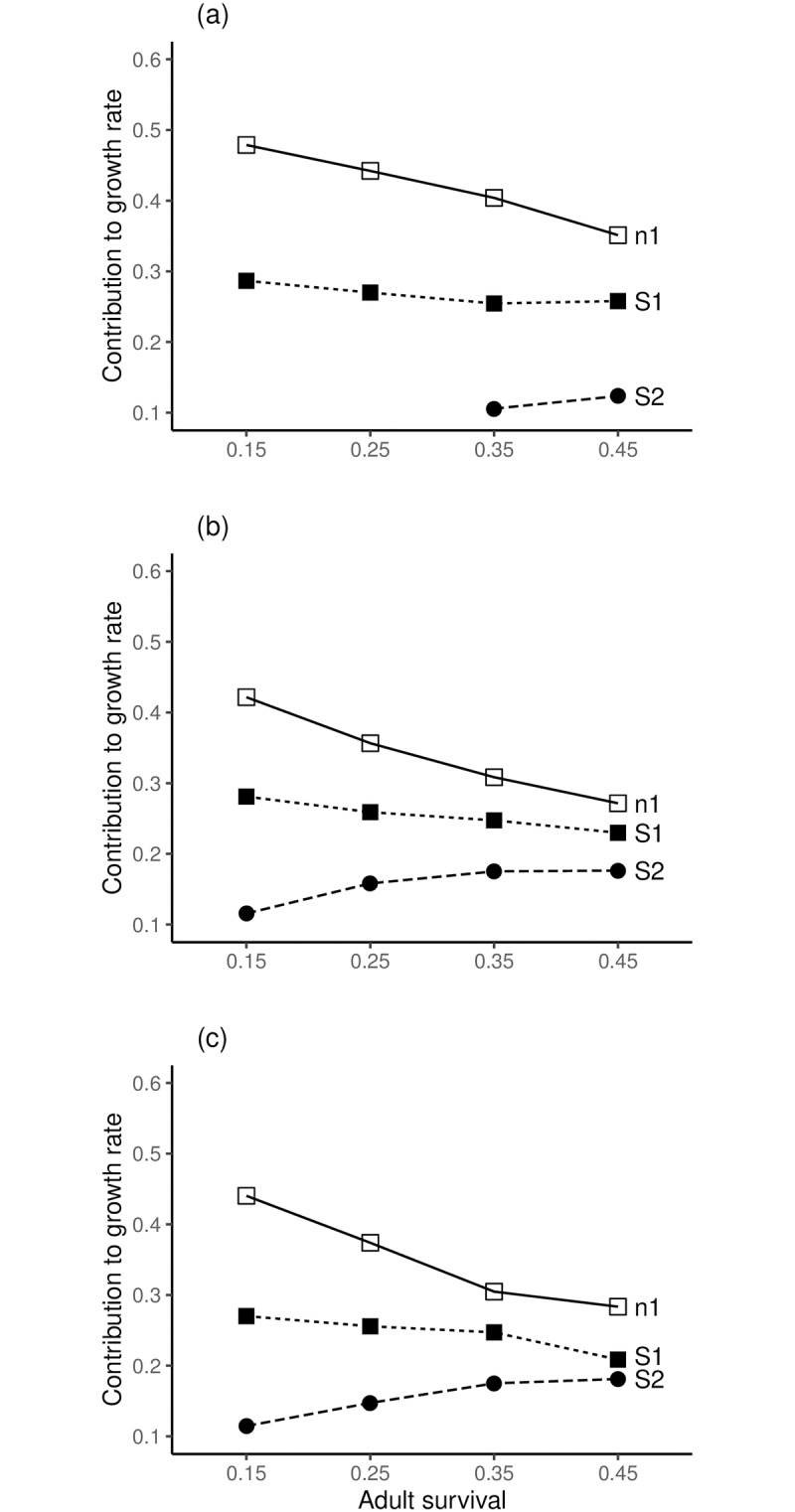
Simulations of age truncation for species with age 1 maturity. (a) *H*. *amarus*, (b) a hypothetical species "average fish", (c) *E*. *motebensis*; proportional contributions exceeding 0.1 of $\sigma_{\lambda t}^{2}$ are shown: *n*_1_ is number of age 1 fish, *S*_1_ and *S*_2_ are survival probabilities for ages 1 and 2, respectively. A bar plot showing the contribution from each model parameter for each transient LTRE is provided in (S4–S6 Figs) for each species.

**Fig 3:**
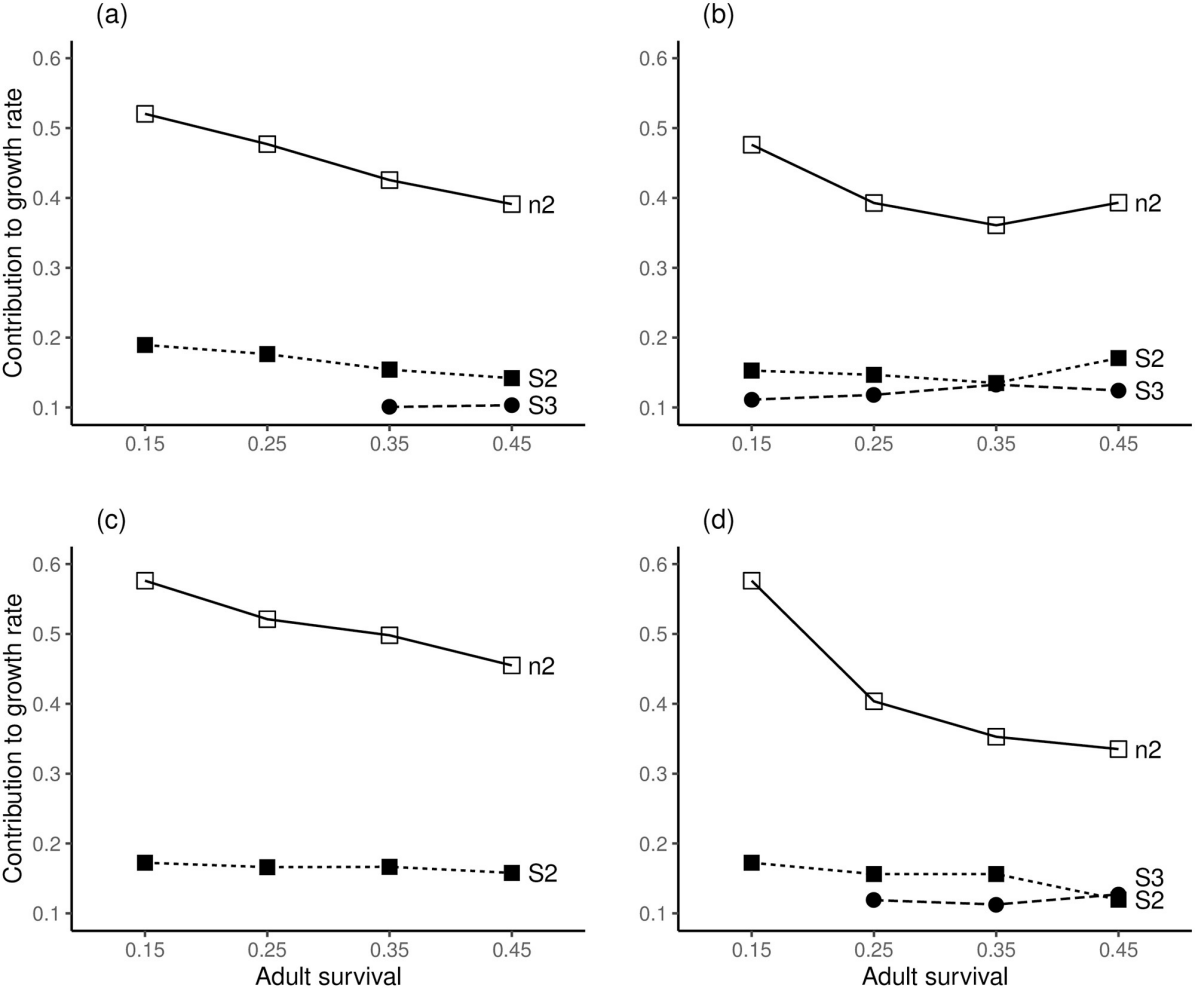
Simulations of age truncation for species with age 2 maturity. (a) a hypothetical species "average fish", (b) *H*. *argyritis*, (c) *P*. *burchelli*, (d) *I*. *lusitanicum*; proportional contributions exceeding 0.1 of $\sigma_{\lambda t}^{2}$ are shown: n_2_ is number of age 2 fish, S_2_, and S_3_ are survival probabilities for ages 2 and 3, respectively. A bar plot showing the contribution from each model parameter for each transient LTRE is provided in (S7–S10 Figs) for each species.

The results of transient LTREs at CV = 0.2 and adult survival values of 0.15, 0.25, 0.35 and 0.45 are summarised by example species and grouped by age 1 or age 2 maturities. Full LTRE results are shown in (S4–S10 Figs). For species with age 1 maturity (Fig 2), the primary drivers of variation in population growth rate are *n*_*1*_, *S*_*1*_ and *S*_*2*_. With reduced adult survival the proportional contributions of *n*_*1*_ and *S*_*1*_ increased whilst that of *S*_*2*_ decreased for all species. The reductions of adult survival to 0.25 and 0.15 represented increases in environmental mortality (*E*) that augment natural mortality (M); respective calculated values of *E* were 0.3365 and 0.8473 with respective survivorship to environmental mortality (*e*^−*E*^) of 0.71 and 0.43. For all example species with age 2 maturity, the main drivers of variation in population growth rate were *n*_*2*_, *S2*, and *S3* (Fig 3) and reduced adult survival similarly increased the proportional contributions to population growth rate from the number and survival of the first reproductive age class. However, the effect of reduced adult survival was greater with delayed maturity (compare Figs 2 and 3). The calculated values of *E* for age 2 maturity at adult survival values of 0.35, 0.25 and 0.15 were 0.2513, 0.5878 and 1.0986, respectively, with survivorship to *E* of 0.78, 0.56 and 0.33, respectively.

## Discussion

Our simulations of a hypothetical "average fish" suggest that iteroparous fishes may share a common risk of endangerment from age truncation in human-altered environments. Across species there appears to be similarity in how drivers of fish population growth respond to age truncation. All the short-lived freshwater species and the hypothetical representations of "average fish" in our simulations responded to age truncation as documented elsewhere for longer-lived and exploited species [21,24]. As adult survival decreased, the number of individuals and survival of the first reproductive age class became progressively more important in determining variation in population growth. The results indicated that population growth rate is driven by a combination of adult survival probability and population structure, especially the number and survival probability of the first reproductive age class. Mean fecundity at-age is not an important contributor to variance of population growth and results are consistent across species in our simulations. These findings agree with observations on 83 fish species of the Yangtze River [9], where juvenile and adult survival were more important than reproductive output. The importance of fecundity for short-lived freshwater fishes has been further questioned recently [41]. Our simulation results suggest that irrespective of their fecundity, short-lived fish species are susceptible to age truncation.

Have the freshwater species in our simulations undergone age truncation? All five example species range in status (IUCN) from near threatened to critically endangered and they share threats from water extractions and exotic predatory species (Table 1). For *H*. *amarus*, Horwitz et al. [42] suggest contemporary adult survival probability from different samples is about 0.1, which is consistent with strong age truncation reducing effective life span from age 4+ to about age 2. Erratic temporal abundance of the species [43] is also consistent with age truncation driving erratic variation in population size [22]. Although we lack sufficient detailed information for the other example species, we suggest it may be possible to detect age truncation in a large sample.

### Caveats and alternate models

With their high biodiversity globally, the rich evolutionary elaboration of fish life histories precludes any single model from accurately representing all species. Details of life history are important for a matrix population model to accurately represent a species’ population growth under specific environmental conditions. We focused narrowly on a systematic exploration of the process of age truncation and comparing simulations within and between species. Our population model mimics an iteroparous life history with reproduction in multiple years after reaching maturity, which is typical for a majority, but not all, freshwater and marine fishes.

There are many possible variations and extensions of our model. For example, fishes with a semelparous life history such as off-shore spawning capelin (*Mallotus villosus*) cannot retain older repeat spawners as represented in our transition matrix, whereas nearshore spawning iteroparous individuals can [44]. As a second example, the effects of reduced adult survival on short-lived marine fish species under commercial exploitation, such as Peruvian anchoveta (*Engraulis ringens*), will require model refinements to simulate its life history. This migratory, pelagic species can spawn throughout the year but exhibits two spawning peaks annually and age classes can be comprised of 2 cohorts a year of different sizes and ages (http://www.fao.org/fishery/species/2917/en, accessed 20 February 2020).

Additional model refinements ought to be examined for individual species in a particular environment. For example, only a fraction of individuals may be reproductively mature at younger adult ages in some species [45–47]. A maturation parameter for the first or several adult age classes could be included in the transition matrix, or, a model of density dependence could be informative with larger body size freshwater fishes. We predict that with partial maturation at age 1, reduced adult survival would yield a population response intermediate to our simulations of age 1 and age 2 maturities. Although we simulate a model of density independence, additional work is needed to clarify how density dependence might affect a population’s response to age truncation.

Environmental perturbations such as river intermittency associated with water extractions or drought, as occurs with four of our five simulated example species, can be included in the population model as a stochastic environmental source of reduced survival. Simulations of alternate models of transient mortality caused by river drying, for example, could be compared to discern the relative importance of juvenile versus adult mortality associated with river intermittency.

### Detecting age truncation

We began our modelling work with *H*. *amarus*, a local species in our region. Initially, we attempted to estimate age-specific adult survival rates (results not shown) with a large sample dataset (S1 Data) but not all were estimable and temporal variation in survival was indicated. This further implied a likely nonequilibrium population structure and conveyed uncertainty about values of survival rates in the contemporary environment. The important question for us became "how might one develop a frame of reference to evaluate contemporary disturbances to a species?". This led to our hypothetical equilibrium baseline for a species in an unperturbed natural environment.

How might a manager detect age truncation in a population? Although manifestation of age truncation may be noticeable through increased fluctuations in population size over time [22], it may be difficult to identify if age truncation is the cause when we only have data on fluctuations in population size. We suggest that a simple binomial proportion (eq. 3) can be calculated on a sufficiently large, unbiased sample and compared with an equilibrium expectation to test for age truncation:

$$\theta_{A}=\left(\sum_{i=2}^{k}n_{i}\right)/\left(\left(\sum_{i=1}^{k}n_{i}\right)\right)$$ (3)

where θ_A_ represents the fraction of adults older than the first reproductive age divided by the total number across all *k* adult ages. The binomial proportion θ_A_ is the converse of Heinke’s method [48]. At the natural equilibrium adult survival rate, and at stable population structure, θ_A_ = 0.349 for an age 4+ lifespan, and θ_A_ = 0.427 for an age 5+ lifespan. The sample data for *H*. *amarus* (S1 Data) yields an estimate of θ_A_ = 0.258 (95% confidence limit: 0.24 ≤ θ_A_ ≤ 0.28), which can be calculated from (S5 Table). In R, the probability of the observed sample given the expected equilibrium value of θ_A_ [*pbinom*(573,2215,0.349)] is 4.1 x 10^−20^, evidence of significant age truncation. We caution that analysis of a single sample should not be construed as proof of age truncation, but rather that the proposed metric θ_A_ should be examined in additional sufficiently large samples over time and space because, for example, an event of strong recruitment to the first reproductive age class will also result in a lower value of θ_A_. Likewise, if strong recruitment events are associated with augmentation of the population from captive production or translocation [6,7], then inference of significant age truncation could be incorrect. Clearly it is important to consider the life span of a species and the contemporary historical (temporal) context for a sample to use θ_A_ as a test of age truncation. For our example species, a simple application of θ_A_ could be to use mean size at age of the second reproductive age class to assign individuals in a sample to first or later adult age classes.

### Identifying causes of age truncation

Although one can test for age truncation, identifying the causes of adult mortality in contemporary environments may be difficult. There are many possible causes of reduced adult survival in fish populations, which may vary across species or across populations of the same species in different environments. As opposed to fishing being a primary cause of age truncation in the marine environment, there are multiple possible contributors to increased adult mortality of freshwater fishes. Direct exploitation of wild populations can drive age truncation for short-lived freshwater fish species in the ornamental fish trade [49]. However, for many fish species, especially those in rivers, causes of reduced adult survival may be indirect, arising from multiple factors and hence more difficult to quantify. The wide-spread introduction of exotic predatory fish species for sport fishing has led to homogenization of freshwater fish communities over large spatial extents [50]. In four of our simulated examples seasonal low river flows are thought to facilitate increased predation mortality by introduced exotic fishes [51–54]. Additionally, over-utilization of freshwater resources and river flow regulation for hydropower or irrigation are important global drivers of population declines in freshwater fish populations [3,55]. For example, water diversions for irrigation can cause seasonal occurrences of river intermittence that dries habitat and kills fish [56]. As a second example, a primary emphasis of conservation for *H*. *amarus* has sought to manage water resources to encourage spawning [43,57] and thereafter habitats can be depleted or dried through irrigation withdrawals. The emphasis on successful spawning involves early season water releases from upstream reservoirs, an irreversible commitment of water resources during drought. This management choice can reduce the water available to promote survival after spawning and it can maintain or increase age truncation if adult mortality occurs with river intermittence. Our results point directly to the importance of adult survival, not fecundity, for population growth of short-lived, iteroparous species like *H*. *amarus*. This implies that during dry years managers should consider reserving water to supplement flows later in the summer when demand is highest for water diversions.

Other human activities may reduce adult survival with indirect and subtle effects because freshwater ecosystems are imbedded in discrete watersheds of the terrestrial landscape. Each watershed is a spatial mosaic of influences from geology, climate and landform coupled with human-mediated ecosystem perturbations through uses of terrestrial and aquatic resources. Landscape impacts to river networks can be cumulative because of the directional topology of rivers. These effects can be subtle but important determinants of species’ distribution and abundance. For example, the spatial extent of agricultural and urban areas in a watershed can be important influences on freshwater invertebrates and fish when analysed with a spatial stream network model [58]. Spatial stream network models [59] offer an important analytic advancement that may facilitate identifying drivers of age truncation in contemporary freshwater environments. One approach, of perhaps several, would be to use θ_A_ as a response variable in a spatial stream network model, where θ_A_ has been estimated at many times and places.

### Implications for biodiversity conservation

Reductions in adult survival are important potential concerns for thousands of fish species that share a common age-structured life history of iteroparity and an indeterminate lifespan dependent on adult survival. Extinction risk increases under age truncation because population persistence becomes more dependent on the first reproductive age class. As a result, population growth becomes highly sensitive to random fluctuations in juvenile survival [22]. For example, an age-truncated population is vulnerable to rapid declines in population size with successive years of lower juvenile survival such as might occur with drought. Managing habitats for increased adult survival would buffer temporal variation in juvenile survival [27] and generally improve reproductive resilience of fish populations. Although captive breeding or translocation [6,7] could be used to accomplish short-term increases in population size, the activity contributes nothing to alleviating the causes of age truncation. To achieve sustainable fisheries we must consider the sensitivity of fish populations to environmental changes that reduce adult survival.

Successful conservation programmes for freshwater biota may depend on accommodating local prevailing cultural and social values [60], which can present significant impediments to ecosystem restoration and biodiversity conservation [56]. A recently proposed emergency recovery plan [61] identified six global action priorities to stem the loss of freshwater biodiversity: (1) implement environmental flows, (2) improve water quality, (3) protect and restore critical habitats, (4) manage exploitation of species and habitats, (5) prevent and control non-native species and (6) safeguard and restore freshwater connectivity. All of these global action priorities are consistent with strategies to alleviate adult mortality in human-altered environments. Of these priorities, provision of environmental flows is fundamental to alleviating causes of age truncation. Although some countries have conferred rights to rivers [61] many others have not. In areas where water is allocated as a property right, such as our local region, there is no incentive to use water efficiently and water resources are insufficient to sustain aquatic systems during many irrigation seasons. Conservation of individual species or restoration of aquatic ecosystems is unlikely to be accomplished without provision of adequate environmental flows water. It has been argued recently that conferring rights to nature, such as rivers and their watersheds, might also directly benefit public health [62]. Alternatively, it has been argued that conferring rights to nature might violate some human rights [63]. Prospects are dim for many freshwater fish species where water has been overallocated to human uses because resolving these social matters will take time, which will likely cause extinction of some species. Conservation of social-ecological freshwater systems requires sustainability of people and ecosystem services that support aquatic biota. When the aquatic biota are endangered, one might logically ask if people and their livelihoods are also at elevated risk.

Sustainability management requires a holistic view of impacts to freshwater ecosystems caused by humans at local to watershed spatial extents and its aim should be managing human uses of land and water in ways that enable restoration of watershed-scale ecological systems [56]. Achieving higher species survival will require paradigm transformations at societal and governmental levels regarding water management priorities and legal accommodations that provide environmental flows of water needed to sustain aquatic ecosystems and their biota [4,61]. Alleviating human impacts to freshwaters is urgently needed for conservation of freshwater biodiversity.

## Supporting information

S1 Table Terms, symbols and definitions. (DOCX)

S2 Table Asymptotic estimates of S_0_. (DOCX)

S3 Table Age 0 survival of *H*. *amarus* in laboratory trials. (DOCX)

S4 Table List of cyprinid species included in Bayesian analysis of fecundity-body length. (DOCX)

S5 Table Mean size at age for *H*. *amarus* collected from the Isleta Reach of the middle Rio Grande of New Mexico in 2009. (DOCX)

S6 Table Results of Bayesian analysis of fecundity-body length. (DOCX)

S1 Fig The general relationships in fishes between life span and (a) M = 4.3/maximum age and (b) S = *e*^*-M*^. (TIF)

S2 Fig Fish fecundity (eggs) and body length data for 33 freshwater species (order Cypriniformes, black symbols, data in S2 Fig) and 342 marine species from 15 orders (gray symbols, data source [30] in main text). (TIF)

S3 Fig Transient LTREs at four levels of coefficient of variation (CV) for survival probabilities: a) CV = 0.05, b) 0.1, c) 0.2 and d) 0.3. (TIF)

S4 Fig Transient LTREs for "average fish", age 1 maturity. (**a**) adult survival = 0.15, (b) 0.25, (c) 0.35 and (d) 0.45.
(TIF)

S5 Fig Transient LTREs for *Enteromius motebensis*, age 1 maturity. (a) adult survival = 0.15, (b) 0.25, (c) 0.35 and (d) 0.45.
(TIF)

S6 Fig Transient LTREs for *Hybognathus amarus*, age 1 maturity. (a) adult survival = 0.15, (b) 0.25, (c) 0.35 and (d) 0.45.
(TIF)

S7 Fig Transient LTREs for "average fish", age 2 maturity. (a) adult survival = 0.15, (b) 0.25, (c) 0.35 and (d) 0.45.
(TIF)

S8 Fig Transient LTREs for *Hybognathus argyritis*, age 2 maturity. (a) adult survival = 0.15, (b) 0.25, (c) 0.35 and (d) 0.45.
(TIF)

S9 Fig Transient LTREs for *Iberochondrostoma lusitanicum*, age 2 maturity. (a) adult survival = 0.15, (b) 0.25, (c) 0.35 and (d) 0.45.
(TIF)

S10 Fig Transient LTREs for *Pseudobarbus burchelli*, age 2 maturity. (a) adult survival = 0.15, (b) 0.25, (c) 0.35 and (d) 0.45.
(TIF)

S1 Data Supporting data. *Sheet 1*. Larval Survival—*H*. *amarus*. *Sheet 2*. Age-length Key—*H*. *amarus*. *Sheet 3*. Fecundity-Length—33 cyprinids. Supporting simulation archive. Dryad, dataset, https://doi.org/10.5061/dryad.69p8cz8z7.
(XLSX)
