# Supplementary material for: Sustainability management of short-lived freshwater fish in human-altered ecosystems should focus on adult survival
Source: PLoS One. 2020 May 12;15(5):e0232872. doi: 10.1371/journal.pone.0232872 (PMC7217442; doi:10.1371/journal.pone.0232872)
Supplement: S1 Table — (DOCX) [file pone.0232872.s001.docx]

**Table S1**. Terms, symbols and definitions

| age truncation | mortality of adults in a population that reduces mean age of breeders and diminishes future reproductive potential, also known as juvenescence or longevity overfishing |
| --- | --- |
| asymptotic | the theoretical demographic conditions attained when vital rates and the environment are stationary |
| *E* | environment-caused mortality that augments natural mortality |
| *F*_i_ | age-specific fecundity, number of eggs |
| iteroparity | an adult reproducing in multiple years |
| λ_1_ | the leading eigenvalue of the transition matrix, the asymptotic population growth rate under stationary conditions |
| λ_t_ | the observed (realised) population growth rate at each time-step in a simulation (λ_t_ = N_t_/N_t-1_) |
| $\sigma_{\lambda t}^{2}$ | variance of realised population growth rate |
| life span | an approximate number of years; it is indeterminate in many fishes because life span depends on adult survival probability |
| LTRE, LTREs | life table response experiment(s) in which vital rates are varied stochastically in simulations and the relative contributions to population growth rate are deduced |
| *M* | natural mortality |
| *n*_i_ | age-specific abundance in a population vector, or the relative abundance when population size is normalized ($\sum n_{i}=1$) |
| normalised | in calculating realised population growth rate, *N*_t-1_ is normalised ($\sum n_{i}=1$) |
| population structure | the relative fraction of the population in each age class |
| predicted fecundity | age-specific fecundity predicted from posterior estimates of a species' intercept and the overall slope in Bayesian meta-analysis of fecundity-body length data |
| retrospective | simulation of a life history and using stochastic values observed to deduce drivers of population growth |
| *S*_i_ | age-specific survival probability of reaching the next age class |
| *S*_E_ | survival rate to environmental mortality, a multiplier of natural mortality |
| stable age distribution | an asymptotic stable condition in population age structure that will be attained when vital rates and the environment are stationary |
| stationary | no change in variation over time |
| θ_A_ | the fraction of adults in the second and later reproductive age classes |
| transient LTRE | a transient life table response experiment in which the variance of λ_t_ is decomposed into fractions attributable to each parameter in the matrix population model; a retrospective analysis of simulated vital rate variation |
| transient variation | random variation in vital rates caused by fluctuations in the environment |
| vital rates | age-specific survival rates and age-specific fecundities |
