# Supplementary material for: Sustainability management of short-lived freshwater fish in human-altered ecosystems should focus on adult survival
Source: PLoS One. 2020 May 12;15(5):e0232872. doi: 10.1371/journal.pone.0232872 (PMC7217442; doi:10.1371/journal.pone.0232872)
Supplement: S2 Table — (DOCX) [file pone.0232872.s002.docx]

**Table S3** Age 0 survival of *H. amarus* in laboratory microcosms.

|  | **Survival Probability** | |
| --- | --- | --- |
| **Microcosm** | **Mean Daily**^†^ | $\boldsymbol{S}_{\boldsymbol{0}}$ |
| A | 0.9797 | 5.610E-4 |
| B | 0.9942 | 1.197E-1 |
| C | 0.9945 | 1.336E-1 |
| D | 0.9936 | 9.599E-2 |
| **Geometric Mean** |  |  |
| A, B, C, D | 0.9905 | 3.046E-2 |
|  | | |

^†^value shown is the geometric mean of n=57 values of daily survival rate for each microcosm

The number of live *H. amarus* was counted daily in each of four microcosms (38 L, filled with 30 L water). Aerated well water maintained at 20˚C, a salinity of 0.4 ppt and propagation in an environmental chamber with a 12h light:12h dark photoperiod approximated ambient river conditions. Juvenile *H. amarus* were fed nauplii of brine shrimp (*Artemia* *salina*). To initiate replicates, fertilized eggs were obtained from eight captive parental pairs that were injected with carp pituitary extract to induce spawning. Eggs were transported to an environmental chamber on day 1 and 700 randomly selected eggs were added to each microcosm. Egg hatching occurred two to three days after fertilization; our estimate of daily survival in each microcosm began with the number of larvae counted on day 4. Daily counts of survival up to day 58 post-hatch were used to calculate daily survival for each microcosm. Data are available in Supporting Information (File S2).
