# Supplementary material for: Sustainability management of short-lived freshwater fish in human-altered ecosystems should focus on adult survival
Source: PLoS One. 2020 May 12;15(5):e0232872. doi: 10.1371/journal.pone.0232872 (PMC7217442; doi:10.1371/journal.pone.0232872)
Supplement: S4 Table — (DOCX) [file pone.0232872.s004.docx]

**Table S4** List of cyprinid species included in Bayesian analysis of fecundity-length.

| **Species** | **n** | **Reference** |
| --- | --- | --- |
| *Alburnoides bipunctatus* | 13 | Polacik, M, Kovác,V. Folia Zool. 2006;55:399-410 |
| *Alburnoides bipunctatus* | 39 | Patimar, R et al. Turk J Zool. 2012; 36:383-393 |
| *Alburnoides sp.* | 22 | Seifali, M et al. Iran J Sci Technol. 2012;A2:181-187 |
| *Alburnus chalcoides* | 188 | Patimar, R et al. Turk J Fish Aquat Sci. 2011;10: 277-285 |
| *Barbus strumicae* | 19 | Sapounidis, A et al. NW J Zool. 2015;11:331-341 |
| *Enteromius humilis* | 19 | Dejen, E et al. Neth J Zool. 2003;52:281-299 |
| *Enteromius motebensis*^a^ | 26 | Kindler, D. MSc Thesis, U Johannesburg. 2015 |
| *Enteromius tanapelagius* | 20 | Dejen, E et al. Neth J Zool. 2003;52:281-299 |
| *Pethia pookodensis* | 37 | Jacob, E. PhD Thesis, Mahatma Ghandi U. 2013 |
| *Pethia ticto* | 59 | Hossain, M et al. J Appl Ichthyol. 2017; doi:10.1111/jai.13427 |
| *Pseudobarbus burchelli* | 17 | Cambray, J, Stuart, C. S Afr J Zool. 1985;20:155-165 |
| *Puntius sophore* | 74 | Hossain, M et al. J. Appl Ichthyol. 2012;28:818-822 |
| *Schizothorax plagiostomus* | 30 | Jan, M et al. J Threat Taxa. 2014;6:5375-5379 |
| *Sahyadria denisonii* | 11 | Solomon, S et al. J Threat Taxa. 2011;3:2071-2077 |
| *Capoeta trutta* | 140 | Patimar, R, Farzi. S. Folia Zool. 2011;60:153-158 |
| *Carassius auratus* | 20 | Amin, R et al. J Agric Vet Sci. 2013;3:36-41 |
| *Carassius gibelio* | 113 | Tarkan, A et al. J Freshw Ecol. 2007;22:11-17 |
| *Labeo horie* | 66 | Dadebo, E et al. Afr J Ecol. 2003;41:31-38 |
| *Chondrostoma regium* | 65 | Mahboobi Soofiani, N et al. Iran J Fish Sci. 2014;13:810-822 |
| *Chrosomus tennesseensis* | 19 | Hamed, M et al. Am Midl Nat. 2008;160:289-299 |
| *Dionda argentosa* | 23 | McMillan, S MSc Thesis, Tx St U-San Marcos. 2011 |
| *Dionda diaboli* | 35 | McMillan, S MSc Thesis, Tx St U-San Marcos. 2011 |
| *Hybognathus placitus* | 28 | Taylor, C. MSc Thesis, Oklahoma State U. 1988 |
| *Hybognathus argyritis* | 11 | Young, J, Koops, M. Can Sci Advis Secretariat, Doc. 2013/084, Ottawa: 2013 |
| *Hybognathus amarus* | 20 | Caldwell, C et al. N Am J Aquac. 2019;81:47-54 |
| *Iberochondrostoma lusitanicum* | 22 | Magalhaes, M et al. J Fish Biol. 2003;63:300-317 |
| *Notropis rafinesquei* | 50 | Haag, W et al. Am Midl Nat. 2007;158:306-320 |
| *Notropis simus pecosensis* | 13 | Hatch (unpubl. data, see Supporting Data File S2) |
| *Phoxinus phoxinus* | 19 | Mills, C, Elorant, Ann Zool Fenn. 1985;22:1-12 |
| *Rhinichthys cataractae* | 18 | Roberts, J, Grossman, G. Ecol Freshw Fish. 2001;10:184-190 |
| *Rhinichthys cobitis* | 14 | Britt, K MSc Thesis, New Mexico State U. 1982 |
| *Rutilus kutum* | 48 | Keivany, Y et al. Res Zool. 2012;2:7-14 |
| *Squalius squalus* | 25 | Lorenzoni, M et al. Knowl Manag Aquat Ecosyst 2011;403:09,doi:10.1051/kmae/2011069 |
| *Squalius torgalensis* | 27 | Magalhaes, M et al. J Fish Biol. 2003;63:300-317 |
