# Supplementary material for: Sustainability management of short-lived freshwater fish in human-altered ecosystems should focus on adult survival
Source: PLoS One. 2020 May 12;15(5):e0232872. doi: 10.1371/journal.pone.0232872 (PMC7217442; doi:10.1371/journal.pone.0232872)
Supplement: S5 Table — (DOCX) [file pone.0232872.s005.docx]

**Table S5** Mean size at age for *H. amarus* collected from the Isleta Reach of the middle Rio Grande of New Mexico in 2009.

| **Age** | **n** | **SL^a^** | **StdDev** | **StdErr** |
| --- | --- | --- | --- | --- |
| 0 | 222 | 43.9 | 2.639 | 0.177 |
| 1 | 1642 | 51.3 | 3.405 | 0.084 |
| 2 | 173 | 61.3 | 2.492 | 0.189 |
| 3 | 241 | 68.8 | 2.530 | 0.163 |
| 4 | 148 | 73.7 | 2.735 | 0.225 |
| 5 | 11 | 81.2 | 1.888 | 0.569 |

^a^mean standard length (mm)

To develop an age-length key for *H. amarus* we used an aged sample comprised of 12 observations of standard length (SL) and age estimated from scale annuli from specimens collected in 1874 [1], augmented by two additional observations from the 2009 sample data. For age 0, we included a SL of 37 mm, which was in the left tail of the frequency distribution of the 2009 sample. For age 1, we included a SL of 52 mm, which was the highest and first peak in the frequency distribution of the 2009 sample data. We fitted a multinomial logistic regression model [2] on 5 mm length intervals of the aged sample using 'nnet' [3] in R [4]. We estimated age [5] for each fish in the sample using the modelled age-length key and the 'alkIndivAge' function in package 'FSA' in R [6](Ogle, 2016). The total fish assigned to each age class was obtained and used to calculate mean length at age. Mean size-at-age values were used to calculate age-specific fecundity values for simulations of age truncation; values are given in Table 2 of the main text. Notice that with this large sample it is not possible to calculate a valid estimate of all age-specific survival probabilities ($n_{i+1}/n_{i}$). Data and an R script are included in File S3.
