# Supplementary material for: Sustainability management of short-lived freshwater fish in human-altered ecosystems should focus on adult survival
Source: PLoS One. 2020 May 12;15(5):e0232872. doi: 10.1371/journal.pone.0232872 (PMC7217442; doi:10.1371/journal.pone.0232872)
Supplement: S6 Table — (DOCX) [file pone.0232872.s006.docx]

**Table S6**. Results of Bayesian analysis of fecundity-body length.

| **Species** | **Estimate** | **SD** | **2.5%** | **97.5%** | **System** | **Rank** |
| --- | --- | --- | --- | --- | --- | --- |
| - "average fish" - |  |  |  |  |  |  |
| overall slope | 3.447 | 0.030 | 3.388 | 3.506 |  |  |
| mean intercept | -8.101 | 0.202 | -8.495 | -7.711 |  |  |
| - species-specific intercepts - |  |  |  |  |  |  |
| *Acanthochromis polyacanthus* | -10.758 | 0.182 | -11.12 | -10.401 | Mar. | 117 |
| *Alburnoides bipunctatus* | -7.547 | 0.141 | -7.819 | -7.271 | Fre. | 37 |
| *Alburnoides sp.* | -8.152 | 0.162 | -8.47 | -7.834 | Fre. | 65 |
| *Alburnus chalcoides* | -9.221 | 0.16 | -9.53 | -8.907 | Fre. | 97 |
| *Amblygaster sirm* | -7.527 | 0.176 | -7.87 | -7.176 | Mar. | 34 |
| *Aphanopus carbo* | -11.66 | 0.243 | -12.134 | -11.191 | Mar. | 118 |
| *Archosargus rhomboidalis* | -6.365 | 0.175 | -6.705 | -6.022 | Mar. | 9 |
| *Atherina presbyter* | -7.581 | 0.156 | -7.89 | -7.274 | Mar. | 40 |
| *Balistes capriscus* | -6.621 | 0.177 | -6.962 | -6.273 | Mar. | 14 |
| *Barbus strumicae* | -9.774 | 0.188 | -10.138 | -9.409 | Fre. | 108 |
| *Canthigaster valentini* | -6.689 | 0.13 | -6.942 | -6.433 | Mar. | 15 |
| *Capoeta trutta* | -9.691 | 0.166 | -10.011 | -9.364 | Fre. | 106 |
| *Carassius auratus* | -6.76 | 0.175 | -7.104 | -6.419 | Fre. | 18 |
| *Carassius gibelio* | -9.098 | 0.169 | -9.426 | -8.766 | Fre. | 95 |
| *Caulolatilus microps* | -8.218 | 0.202 | -8.606 | -7.825 | Mar. | 68 |
| *Centropomus undecimalis* | -8.296 | 0.231 | -8.752 | -7.843 | Mar. | 71 |
| *Cephalopholis cruentata* | -6.575 | 0.204 | -6.974 | -6.174 | Mar. | 12 |
| *Chondrostoma regium* | -9.052 | 0.156 | -9.353 | -8.743 | Fre. | 94 |
| *Chrosomus tennesseensis* | -7.006 | 0.15 | -7.297 | -6.712 | Fre. | 23 |
| *Cynoscion regalis* | -8.579 | 0.208 | -8.985 | -8.165 | Mar. | 83 |
| *Dionda argentosa* | -9.447 | 0.154 | -9.75 | -9.146 | Fre. | 101 |
| *Dionda diaboli* | -8.935 | 0.139 | -9.205 | -8.662 | Fre. | 93 |
| *Elagatis bipinnulata* | -8.39 | 0.212 | -8.804 | -7.976 | Mar. | 72 |
| *Engraulis anchoita* | -7.809 | 0.154 | -8.111 | -7.509 | Mar. | 52 |
| *Engraulis mordax* | -7.015 | 0.169 | -7.348 | -6.683 | Mar. | 24 |
| *Engraulis ringens* | -7.746 | 0.156 | -8.048 | -7.438 | Mar. | 48 |
| *Enteromius humilis* | -7.426 | 0.16 | -7.737 | -7.109 | Fre. | 29 |
| **Enteromius motebensis* | -6.958 | 0.15 | -7.252 | -6.663 | Fre. | 21 |
| *Enteromius tanapelagius* | -8.071 | 0.158 | -8.371 | -7.762 | Fre. | 64 |
| *Epinephelus aeneus* | -7.765 | 0.209 | -8.18 | -7.354 | Mar. | 51 |
| *Ethmalosa fimbriata* | -8.804 | 0.183 | -9.165 | -8.441 | Mar. | 89 |
| *Gadus morhua* | -8.559 | 0.204 | -8.957 | -8.156 | Mar. | 82 |
| *Gasterosteus aculeatus* | -8.513 | 0.126 | -8.756 | -8.264 | Mar. | 78 |
| *Genyonemus lineatus* | -9.242 | 0.172 | -9.573 | -8.9 | Mar. | 98 |
| *Hippoglossoides platessoides* | -7.262 | 0.166 | -7.581 | -6.937 | Mar. | 27 |
| *Hoplostethus atlanticus* | -9.883 | 0.181 | -10.234 | -9.526 | Mar. | 111 |
| **Hybognathus amarus* | -6.041 | 0.159 | -6.353 | -5.729 | Fre. | 5 |
| *Hybognathus placitus* | -7.489 | 0.148 | -7.779 | -7.2 | Fre. | 32 |
| **Hybognthus argyritis* | -7.054 | 0.189 | -7.423 | -6.685 | Fre. | 25 |
| **Iberochondrostoma lusitanicum* | -7.556 | 0.161 | -7.87 | -7.243 | Fre. | 38 |
| *Labeo horie* | -9.419 | 0.202 | -9.816 | -9.027 | Fre. | 99 |
| *Larimus fasciatus* | -5.229 | 0.154 | -5.526 | -4.925 | Mar. | 2 |
| *Lates calcarifer* | -7.615 | 0.223 | -8.047 | -7.18 | Mar. | 43 |
| *Lutjanus carponotatus* | -7.456 | 0.173 | -7.795 | -7.117 | Mar. | 30 |
| *Mallotus villosus* | -7.862 | 0.167 | -8.184 | -7.538 | Mar. | 58 |
| *Merlangius merlangus* | -6.838 | 0.177 | -7.182 | -6.493 | Mar. | 20 |
| *Merluccius hubbsi* | -8.601 | 0.192 | -8.974 | -8.226 | Mar. | 84 |
| *Merluccius merluccius* | -9.834 | 0.193 | -10.209 | -9.455 | Mar. | 109 |
| *Micropogonias furnieri* | -8.861 | 0.202 | -9.251 | -8.471 | Mar. | 92 |
| *Mugil cephalus* | -6.277 | 0.175 | -6.615 | -5.934 | Mar. | 8 |
| *Notropis rafinesquei* | -7.474 | 0.122 | -7.711 | -7.238 | Fre. | 31 |
| *Notropis simus pecosensis* | -6.965 | 0.17 | -7.3 | -6.635 | Fre. | 22 |
| *Odontesthes argentinensis* | -9.926 | 0.187 | -10.286 | -9.554 | Mar. | 112 |
| *Oxylebius pictus* | -7.598 | 0.176 | -7.941 | -7.257 | Mar. | 42 |
| *Paralichthys dentatus* | -7.414 | 0.192 | -7.782 | -7.038 | Mar. | 28 |
| *Paralichthys patagonicus* | -10.066 | 0.206 | -10.464 | -9.657 | Mar. | 114 |
| *Paralonchurus brasiliensis* | -7.949 | 0.193 | -8.329 | -7.571 | Mar. | 61 |
| *Pethia pookodensis* | -6.725 | 0.135 | -6.986 | -6.456 | Fre. | 17 |
| *Pethia ticto* | -5.708 | 0.135 | -5.97 | -5.442 | Fre. | 4 |
| *Phoxinus phoxinus* | -8.832 | 0.156 | -9.139 | -8.529 | Fre. | 90 |
| *Planiliza subviridis* | -5.253 | 0.187 | -5.623 | -4.891 | Mar. | 3 |
| *Pleuronectes platessa* | -8.785 | 0.182 | -9.138 | -8.424 | Mar. | 88 |
| *Pomacentrus coelestis* | -5.057 | 0.124 | -5.299 | -4.815 | Mar. | 1 |
| *Pomatoschistus minutus* | -6.717 | 0.151 | -7.012 | -6.424 | Mar. | 16 |
| **Pseudobarbus burchelli* | -7.857 | 0.171 | -8.194 | -7.523 | Fre. | 56 |
| *Pseudopleuronectes americanus* | -6.57 | 0.203 | -6.966 | -6.17 | Mar. | 11 |
| *Puntius sophore* | -6.266 | 0.137 | -6.531 | -5.998 | Fre. | 7 |
| *Reinhardtius hippoglossoides* | -12.202 | 0.201 | -12.591 | -11.804 | Mar. | 119 |
| *Rhinichthys cataractae* | -8.427 | 0.165 | -8.745 | -8.11 | Fre. | 74 |
| *Rhinichthys cobitis* | -8.845 | 0.164 | -9.167 | -8.517 | Fre. | 91 |
| *Rhomboplites aurorubens* | -8.249 | 0.194 | -8.624 | -7.867 | Mar. | 69 |
| *Rutilus kutum* | -9.85 | 0.195 | -10.228 | -9.465 | Fre. | 110 |
| *Sahyadria denisonii* | -9.44 | 0.189 | -9.804 | -9.066 | Fre. | 100 |
| *Sardinops sagax* | -8.034 | 0.157 | -8.339 | -7.728 | Mar. | 62 |
| *Schizothorax plagiostomus* | -10.68 | 0.19 | -11.047 | -10.308 | Fre. | 116 |
| *Scomber scombrus* | -7.22 | 0.196 | -7.599 | -6.833 | Mar. | 26 |
| *Scomberomorus cavalla* | -8.75 | 0.213 | -9.161 | -8.324 | Mar. | 87 |
| *Sebastes alutus* | -9.577 | 0.183 | -9.934 | -9.215 | Mar. | 102 |
| *Sebastes atrovirens* | -7.542 | 0.201 | -7.934 | -7.148 | Mar. | 36 |
| *Sebastes auriculatus* | -8.545 | 0.194 | -8.919 | -8.163 | Mar. | 80 |
| *Sebastes carnatus* | -7.625 | 0.213 | -8.049 | -7.213 | Mar. | 44 |
| *Sebastes caurinus* | -8.49 | 0.19 | -8.858 | -8.121 | Mar. | 76 |
| *Sebastes chlorostictus* | -7.761 | 0.18 | -8.109 | -7.411 | Mar. | 50 |
| *Sebastes constellatus* | -7.945 | 0.195 | -8.327 | -7.564 | Mar. | 60 |
| *Sebastes crameri* | -8.718 | 0.219 | -9.143 | -8.292 | Mar. | 86 |
| *Sebastes dallii* | -7.823 | 0.17 | -8.155 | -7.491 | Mar. | 53 |
| *Sebastes diploproa* | -7.914 | 0.202 | -8.304 | -7.519 | Mar. | 59 |
| *Sebastes elongatus* | -7.84 | 0.188 | -8.204 | -7.467 | Mar. | 54 |
| *Sebastes entomelas* | -8.184 | 0.193 | -8.558 | -7.808 | Mar. | 67 |
| *Sebastes flavidus* | -8.152 | 0.195 | -8.53 | -7.773 | Mar. | 66 |
| *Sebastes goodei* | -8.405 | 0.192 | -8.774 | -8.03 | Mar. | 73 |
| *Sebastes hopkinsi* | -8.452 | 0.173 | -8.787 | -8.11 | Mar. | 75 |
| *Sebastes jordani* | -9.214 | 0.213 | -9.629 | -8.8 | Mar. | 96 |
| *Sebastes levis* | -8.614 | 0.211 | -9.03 | -8.204 | Mar. | 85 |
| *Sebastes melanops* | -7.517 | 0.184 | -7.874 | -7.155 | Mar. | 33 |
| *Sebastes mentella* | -10.292 | 0.179 | -10.64 | -9.942 | Mar. | 115 |
| *Sebastes miniatus* | -8.048 | 0.195 | -8.423 | -7.663 | Mar. | 63 |
| *Sebastes mystinus* | -7.749 | 0.175 | -8.093 | -7.414 | Mar. | 49 |
| *Sebastes norvegicus* | -9.607 | 0.192 | -9.977 | -9.228 | Mar. | 105 |
| *Sebastes paucispinis* | -8.558 | 0.198 | -8.941 | -8.171 | Mar. | 81 |
| *Sebastes pinniger* | -7.85 | 0.23 | -8.3 | -7.398 | Mar. | 55 |
| *Sebastes rosaceus* | -7.655 | 0.183 | -8.01 | -7.297 | Mar. | 45 |
| *Sebastes rosenblatti* | -7.709 | 0.178 | -8.058 | -7.358 | Mar. | 47 |
| *Sebastes rufus* | -8.506 | 0.201 | -8.898 | -8.104 | Mar. | 77 |
| *Sebastes saxicola* | -7.581 | 0.171 | -7.915 | -7.247 | Mar. | 41 |
| *Sebastes semicinctus* | -7.859 | 0.166 | -8.183 | -7.534 | Mar. | 57 |
| *Sebastes serranoides* | -8.529 | 0.187 | -8.892 | -8.167 | Mar. | 79 |
| *Sebastes viviparus* | -9.604 | 0.179 | -9.947 | -9.251 | Mar. | 104 |
| *Seriphus politus* | -7.529 | 0.155 | -7.832 | -7.221 | Mar. | 35 |
| *Siganus canaliculatus* | -6.602 | 0.186 | -6.966 | -6.239 | Mar. | 13 |
| *Solea solea* | -7.568 | 0.178 | -7.915 | -7.22 | Mar. | 39 |
| *Spratelloides gracilis* | -6.059 | 0.157 | -6.362 | -5.755 | Mar. | 6 |
| *Squalius squalus* | -9.98 | 0.189 | -10.352 | -9.606 | Fre. | 113 |
| *Squalius torgalensis* | -8.287 | 0.156 | -8.595 | -7.987 | Fre. | 70 |
| *Stegastes fuscus* | -6.378 | 0.171 | -6.71 | -6.041 | Mar. | 10 |
| *Stellifer rastrifer* | -7.682 | 0.168 | -8.013 | -7.356 | Mar. | 46 |
| *Thalassoma bifasciatum* | -6.817 | 0.132 | -7.076 | -6.559 | Mar. | 19 |
| *Thunnus alalunga* | -9.731 | 0.213 | -10.14 | -9.317 | Mar. | 107 |

Each species in our simulations (*) is in the upper one-half of intercepts for all 119 species, and three of the five examples are in the upper one-quarter of relative fecundity. Species' ranks from highest fecundity to lowest were: *H. amarus* (captive stock; 5 of 119), *E. motebensis* (21), *H. argyritis* (25), *I. lusitanicum* (38) and *P. burchelli* (56).
