# Supplementary figures and images for: Sustainability management of short-lived freshwater fish in human-altered ecosystems should focus on adult survival
Source: PLoS One. 2020 May 12;15(5):e0232872. doi: 10.1371/journal.pone.0232872 (PMC7217442; doi:10.1371/journal.pone.0232872)

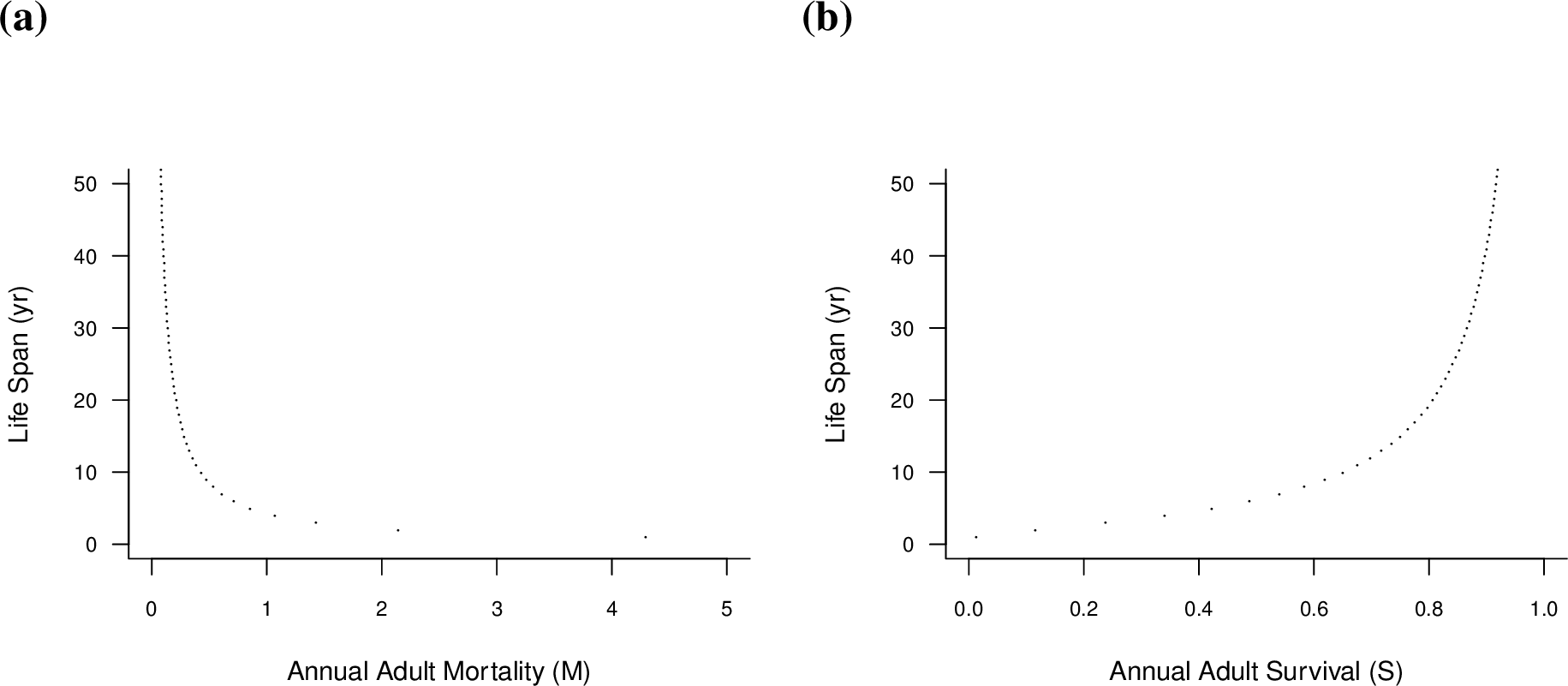

Supplement: S1 Fig — (TIF) [file pone.0232872.s007.tif]

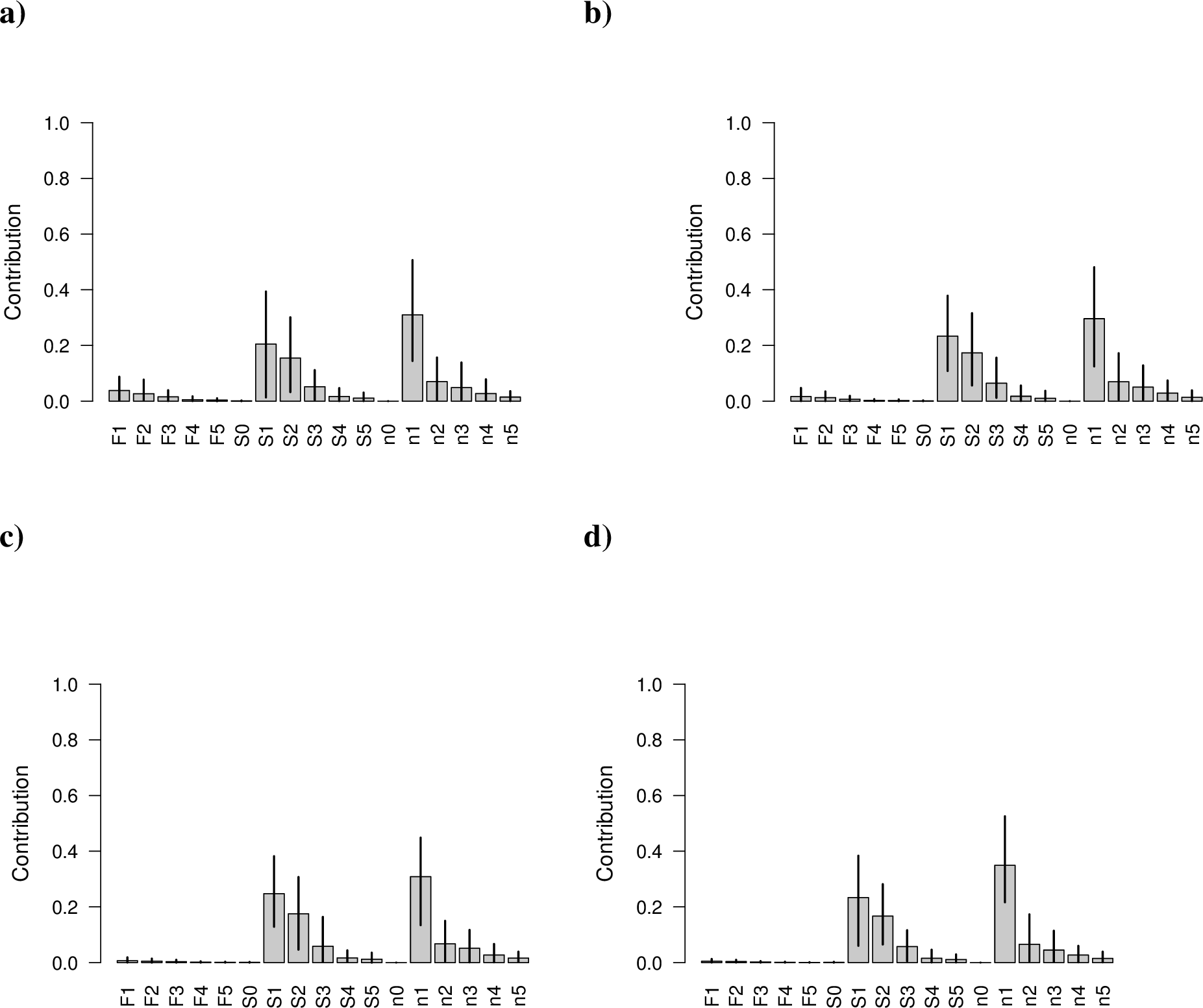

Supplement: S2 Fig — (TIF) [file pone.0232872.s008.tif]

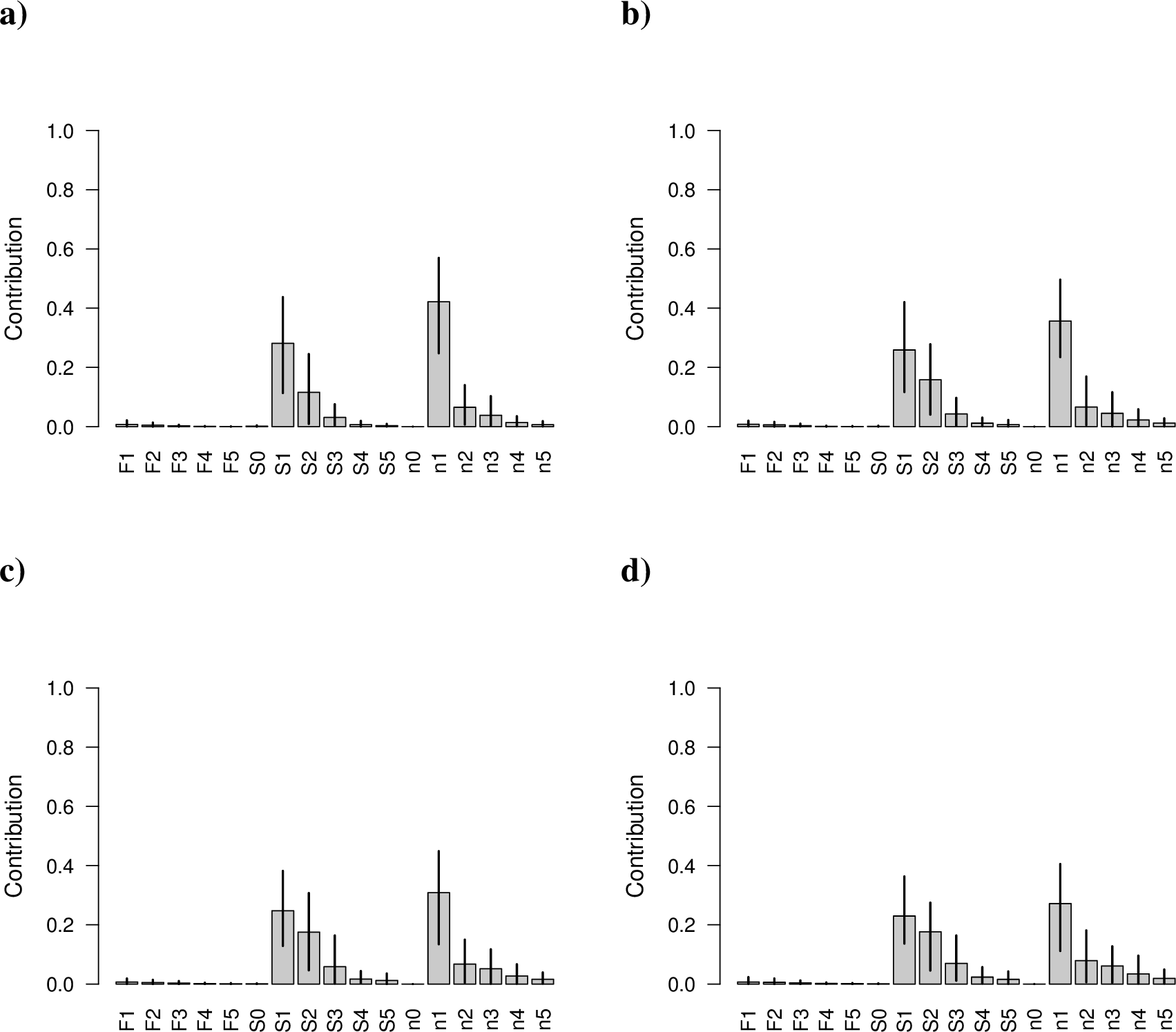

Supplement: S4 Fig — (a) adult survival = 0.15, (b) 0.25, (c) 0.35 and (d) 0.45. (TIF) [file pone.0232872.s010.tif]

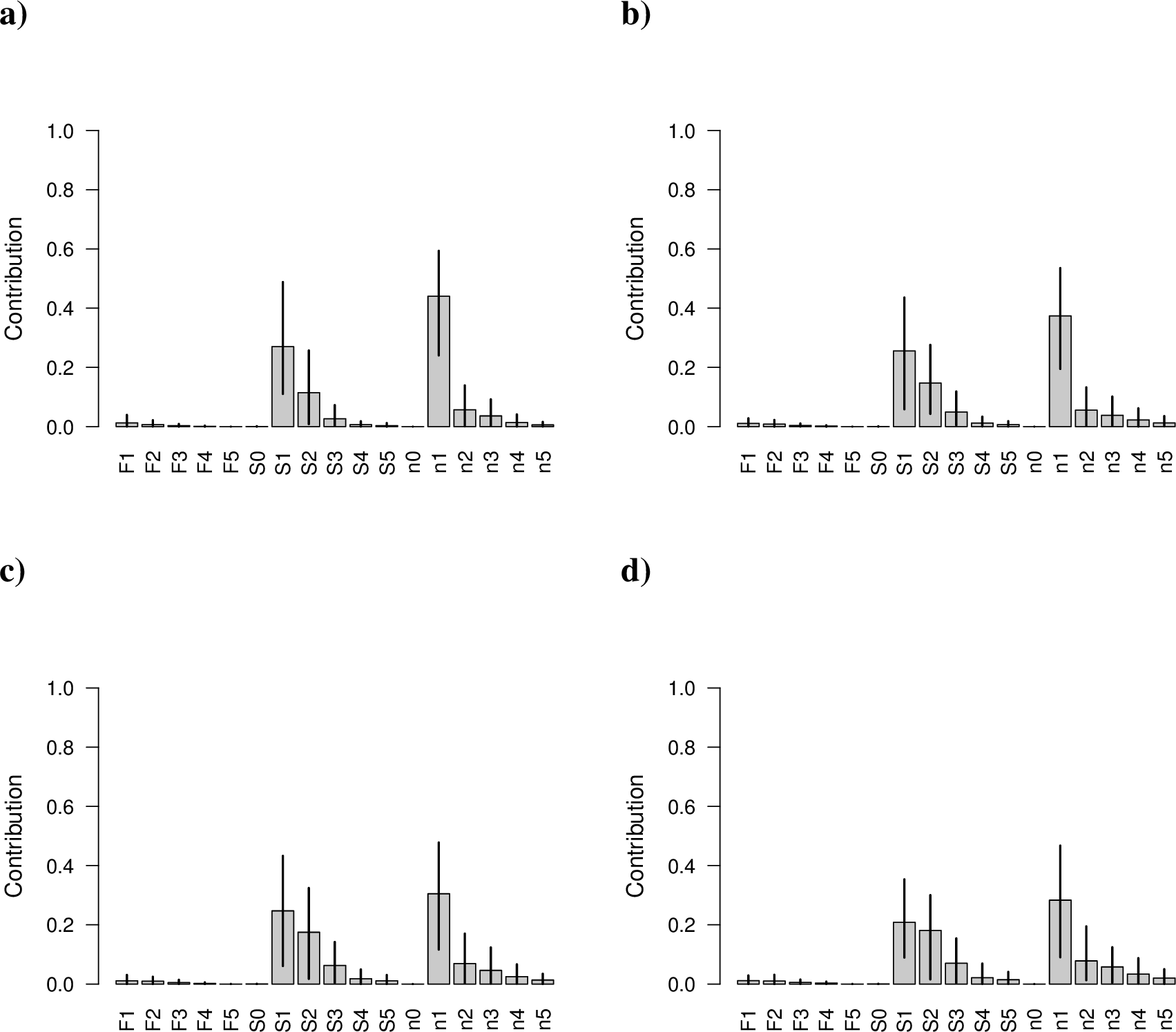

Supplement: S5 Fig — (a) adult survival = 0.15, (b) 0.25, (c) 0.35 and (d) 0.45. (TIF) [file pone.0232872.s011.tif]

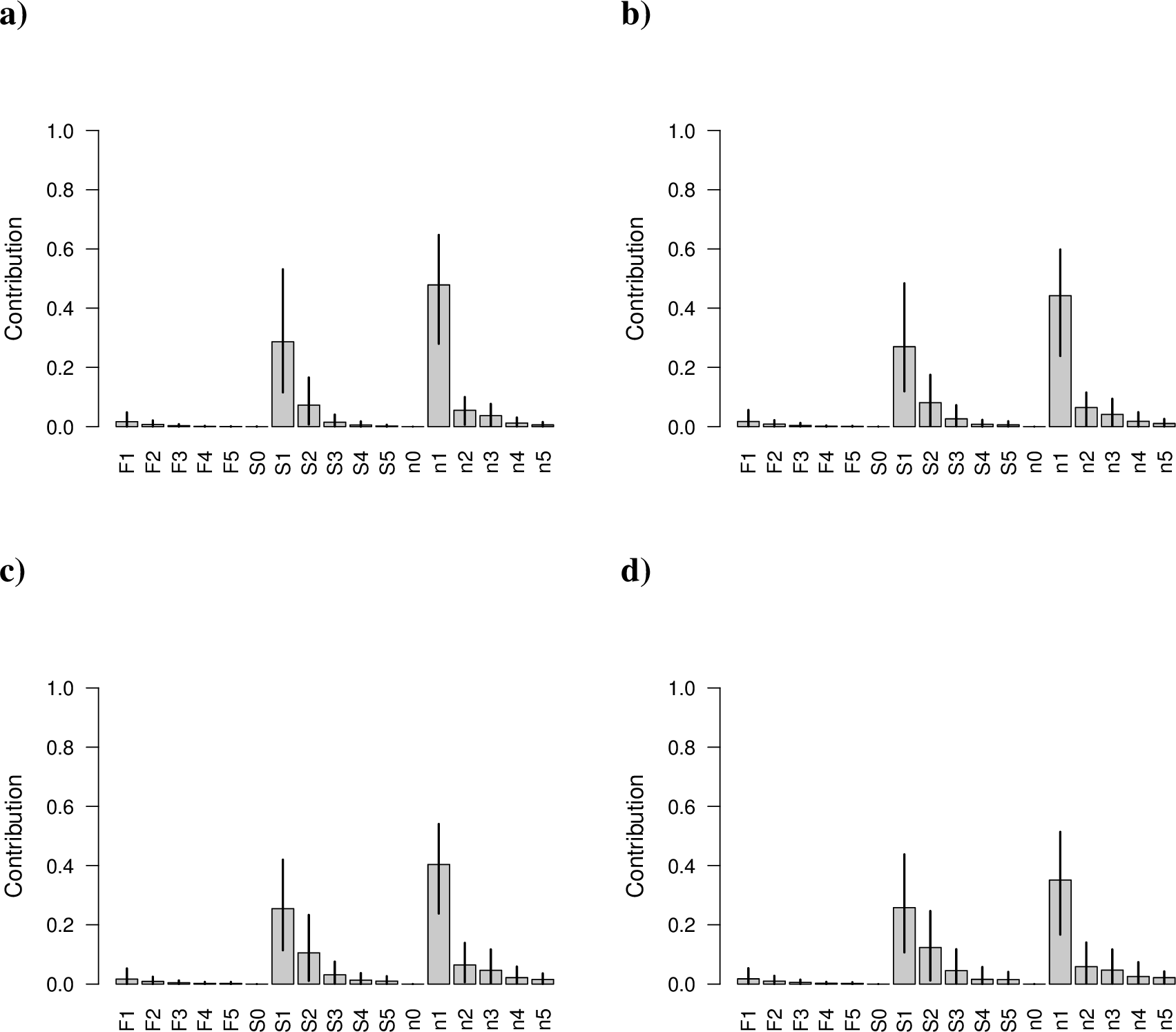

Supplement: S6 Fig — (a) adult survival = 0.15, (b) 0.25, (c) 0.35 and (d) 0.45. (TIF) [file pone.0232872.s012.tif]

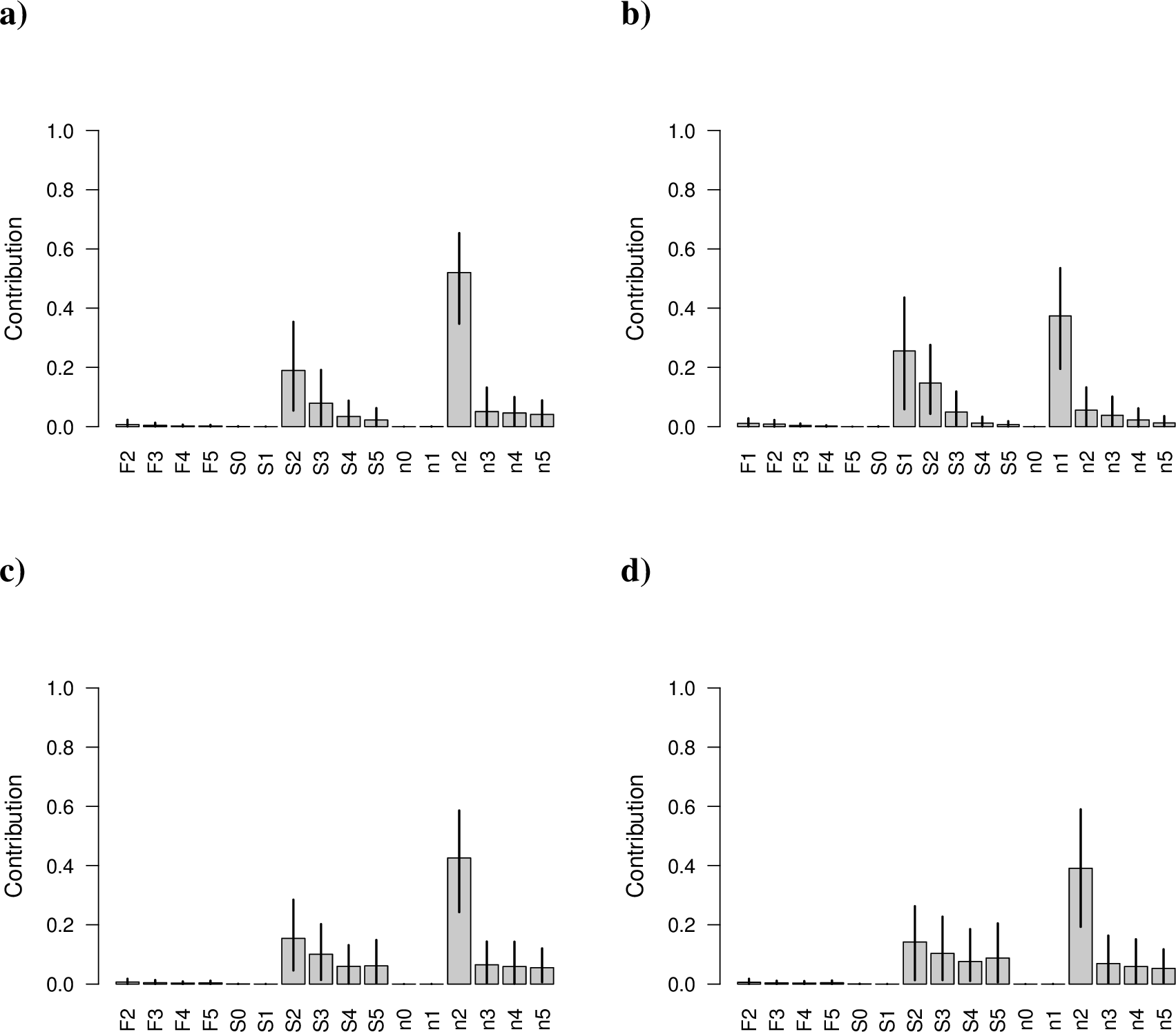

Supplement: S7 Fig — (a) adult survival = 0.15, (b) 0.25, (c) 0.35 and (d) 0.45. (TIF) [file pone.0232872.s013.tif]

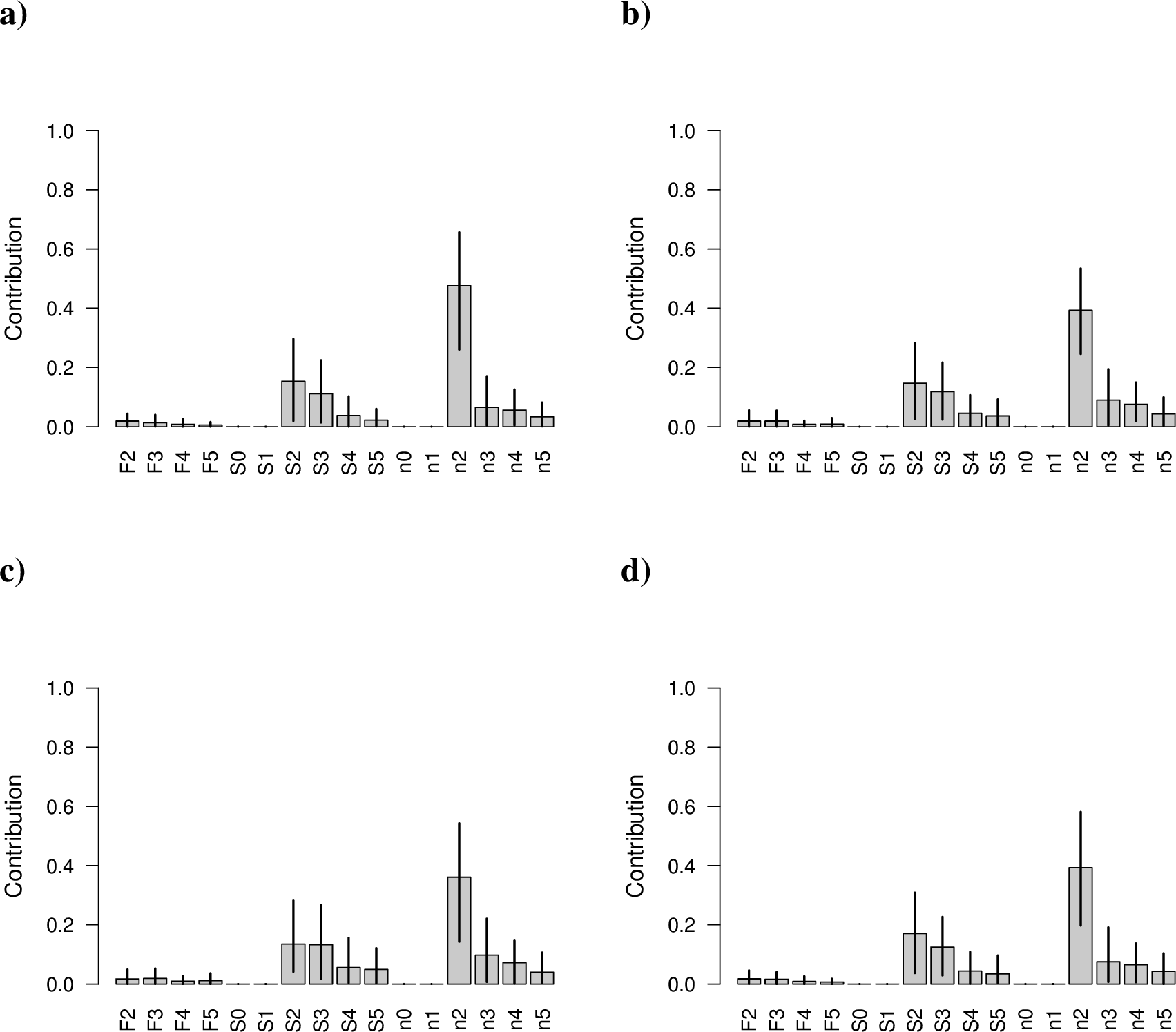

Supplement: S8 Fig — (a) adult survival = 0.15, (b) 0.25, (c) 0.35 and (d) 0.45. (TIF) [file pone.0232872.s014.tif]

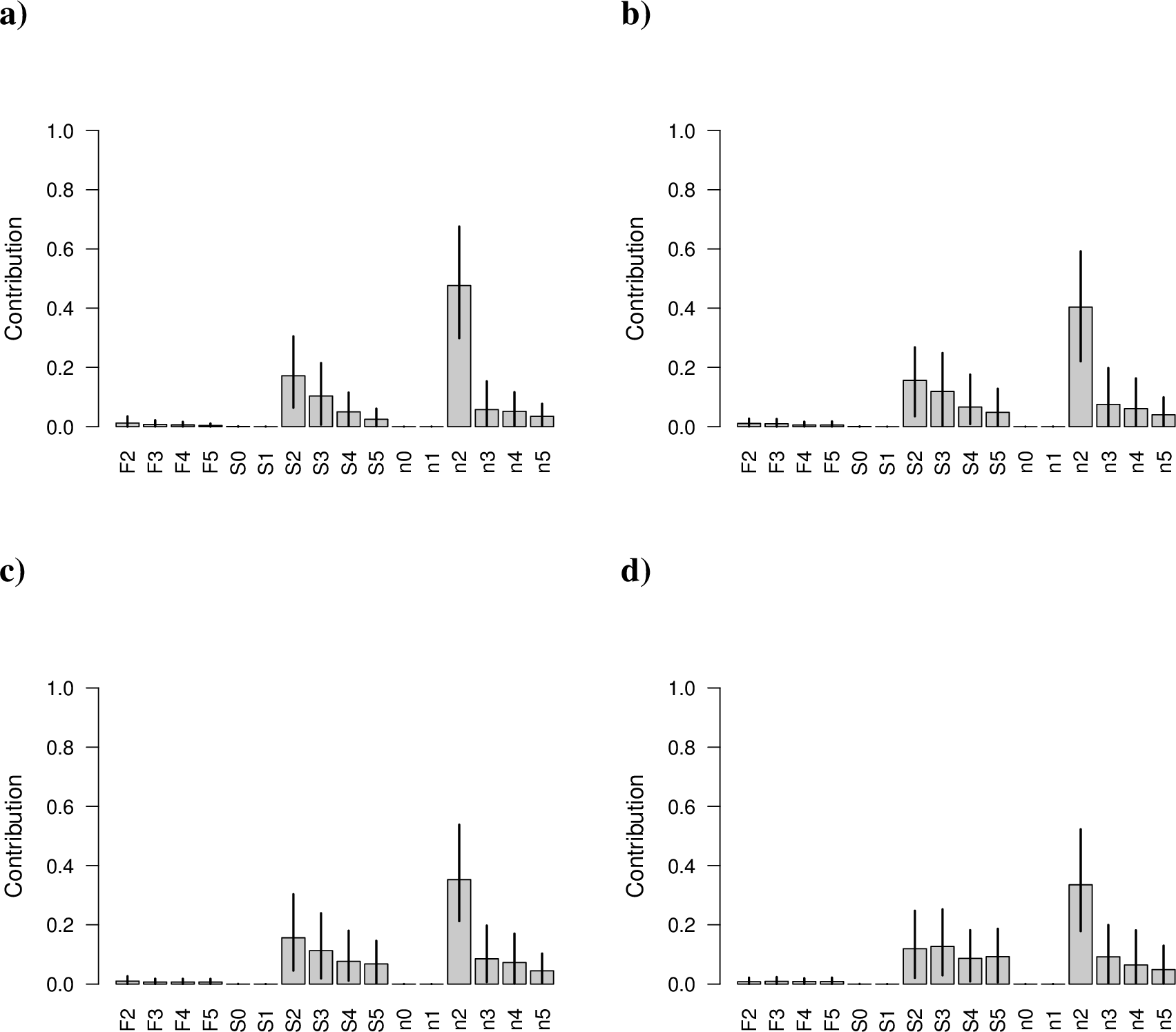

Supplement: S9 Fig — (a) adult survival = 0.15, (b) 0.25, (c) 0.35 and (d) 0.45. (TIF) [file pone.0232872.s015.tif]

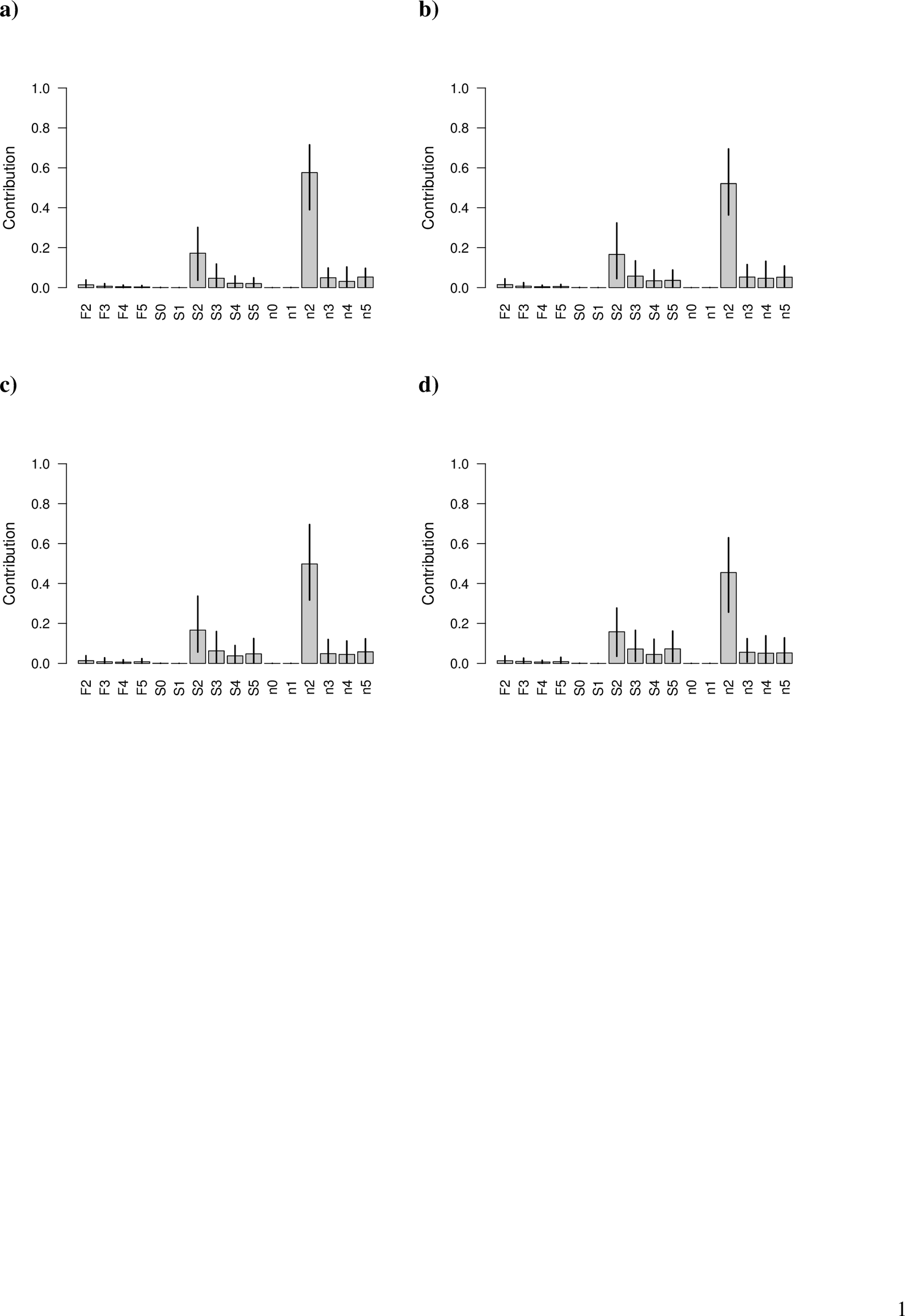

Supplement: S10 Fig — (a) adult survival = 0.15, (b) 0.25, (c) 0.35 and (d) 0.45. (TIF) [file pone.0232872.s016.tif]
